# M-type pyruvate kinase 2 (PKM2) tetramerization alleviates the progression of right ventricle failure by regulating oxidative stress and mitochondrial dynamics

**DOI:** 10.1186/s12967-023-04780-6

**Published:** 2023-12-07

**Authors:** Lizhe Guo, Lu Wang, Gang Qin, Junjie Zhang, Jin Peng, Longyan Li, Xiang Chen, Dandan Wang, Jian Qiu, E. Wang

**Affiliations:** 1 Department of Anesthesiology, Xiangya Hospital, Central South University, Changsha, China; 2 Hunan Key Laboratory of Molecular Precision Medicine, Department of Neurology, Xiangya Hospital, Central South University, Changsha, China; 3 National Clinical Research Center for Geriatric Disorders (Xiangya Hospital), Changsha, China; 4 Hunan Key Laboratory of Medical Genetics, School of Life Sciences, Central South University, Changsha, Hunan China

**Keywords:** Pulmonary arterial hypertension, Right ventricular failure, PKM2 tetramerization, Mitochondrial dynamics, Myocardial apoptosis

## Abstract

**Background:**

Right ventricle failure (RVF) is a progressive heart disease that has yet to be fully understood at the molecular level. Elevated M-type pyruvate kinase 2 (PKM2) tetramerization alleviates heart failure, but detailed molecular mechanisms remain unclear.

**Objective:**

We observed changes in PKM2 tetramerization levels during the progression of right heart failure and in vitro cardiomyocyte hypertrophy and explored the causal relationship between altered PKM2 tetramerization and the imbalance of redox homeostasis in cardiomyocytes, as well as its underlying mechanisms. Ultimately, our goal was to propose rational intervention strategies for the treatment of RVF.

**Method:**

We established RVF in Sprague Dawley (SD) rats by intraperitoneal injection of monocrotaline (MCT). The pulmonary artery pressure and right heart function of rats were assessed using transthoracic echocardiography combined with right heart catheterization. TEPP-46 was used both in vivo and in vitro to promote PKM2 tetramerization.

**Results:**

We observed that oxidative stress and mitochondrial disorganization were associated with increased apoptosis in the right ventricular tissue of RVF rats. Quantitative proteomics revealed that PKM2 was upregulated during RVF and negatively correlated with the cardiac function. Facilitating PKM2 tetramerization promoted mitochondrial network formation and alleviated oxidative stress and apoptosis during cardiomyocyte hypertrophy. Moreover, enhancing PKM2 tetramer formation improved cardiac mitochondrial morphology, mitigated oxidative stress and alleviated heart failure.

**Conclusion:**

Disruption of PKM2 tetramerization contributed to RVF by inducing mitochondrial fragmentation, accumulating ROS, and finally promoted the progression of cardiomyocyte apoptosis. Facilitating PKM2 tetramerization holds potential as a promising therapeutic approach for RVF.

**Supplementary Information:**

The online version contains supplementary material available at 10.1186/s12967-023-04780-6.

## Introduction

Pulmonary arterial hypertension (PAH) describes patients with an average pulmonary artery pressure of > 20 mmHg, characterized by a progressive increase in pulmonary vascular resistance, eventually leading to right ventricular failure (RVF) [1]. The adaptation of right ventricular function to increased afterload depends on the increased contractility by cardiomyocyte hypertrophy to match the increase in arterial elasticity. However, hypertrophic right ventricle will develop into RV–arterial uncoupling, which eventually leads to right ventricular dilation and RVF [2]. Primary pulmonary vascular lesions are believed to be the root cause of right heart failure due to PAH. A large number of studies have been devoted to elucidating the pathogenesis of progressive pulmonary vascular thickening in patients with PAH [3–5]. However, clinical evidence suggests that treating only the pulmonary vascular phenotype could not significantly improve the long-term survival of PAH patients [6]. Even with the improvements in pulmonary hemodynamic status, poor right ventricular function persists in PAH patients and deteriorates the prognosis [7]. Therefore, it is the focus of current research how to alleviate the adverse myocardial remodeling of right heart in PAH patients to eventually improve the prognosis of the disease.

Tumor cells and myocardial cells under pathological conditions both exhibit high expression of M-type pyruvate kinase 2 (PKM2) [8, 9], which catalyzes a rate-limiting step of glycolysis to produce pyruvate and adenosine triphosphate (ATP) from phosphoenolpyruvate and adenosine diphosphate (ADP). PKM2 oscillates among tetrameric, dimeric and monomeric forms [10]. The tetrameric form of PKM2 exerts the enzyme activity of glycolysis [11, 12]. However, in tumor cells, elevated oxidative stress could destabilize PKM2 tetramer through redox modifications on its cysteine residues [8, 10, 13]. Reduced catalytic activity of PKM2 thus diverts glucose metabolism flux towards the pentose phosphate pathway to generate adequate reducing power against reactive oxygen species (ROS), thus facilitating tumor cell proliferation. Oxidative stress and energetic metabolism reprogramming are well documented during the progression of heart failure [14]. Cardiomyocyte apoptosis induced by ROS is the common mechanism of decompensation of left and right heart failure [15]. Interestingly, elevated expression of PKM2 and enhanced tetramerization alleviate heart failure and improve myocardial regeneration [16–19], but detailed molecular mechanisms are incompletely understood.

In the present study, we further investigated the underlying molecular mechanisms through which PKM2 regulates cardiac function during right heart failure and explored whether leveraging PKM2 tetramerization might serve as an effective approach to improve cardiac function.

## Methods and materials

### Animal model

Sprague–Dawley (SD) rats (weighing 220–250 g) from the Department of Laboratory Animals of Central South University (Changsha, China) were employed. All animal experiments were carried out in accordance with the recommendations of national and international animal care and ethical guidelines and were approved by the Ethics Committee for Animal Research of Xiangya Hospital of Central South University (permit code: 2103590). Monocrotaline (MCT, 55 mg/kg; C2401, powder, Sigma-Aldrich) was dissolved in saline containing 20% ethanol, and intraperitoneally injected into SD rats to establish PAH as described previously [20]. Animal experiments were performed in three batches with 5–6 rats per group (either the control group with solvent and the RVF group, or the RVF group with solvent and the RVF + TEPP-46 group). All rats were housed at 25 ± 2 °C with a 12-h light–dark cycle and free access to food and water. Transthoracic echocardiography was performed every 7 days to measure the maximum velocity of tricuspid regurgitation before day 21, and every 2 days after day 21. A maximum tricuspid regurgitation velocity greater than or equal to 2.5 m/s was the criterion for successful establishment of the PAH model. Right ventricular function was determined by measuring the fractional area change (FAC), the ratio between the difference in end-diastolic and end-systolic area and the end-diastolic area of the right ventricle. Right ventricular tissues were harvested at day 28. At the end of transthoracic echocardiography, the rats were euthanized with anesthetics, the heart tissues were collected. The rest parts of the body were bio-safely disposed by the Department of Laboratory Animals of Central South University.

### Analysis of right ventricular systolic pressure and cardiac function

We used transthoracic echocardiography to collect and analyze right ventricular systolic pressure and function data in rats as previously described [20]. All rats were anaesthetized by inhalation of 6% to 8% (v/v) sevoflurane, and anaesthesia was maintained with 2% (v/v) sevoflurane. All rats were placed on a heating pad to maintain a body temperature of 37 °C. Echocardiography was performed with the Vivid E7 system (General Electric Vingmed). Right ventricular systolic pressure (RVSP) was equal to cross-valve pressure plus an estimated right atrial pressure (RAP) of 10 mmHg. Cross-valve pressure was estimated by calculating the maximum velocity of the tricuspid regurgitant (TRmax) jet using the modified Bernoulli equation. Right ventricular function was determined by measuring the fractional area change (FAC), the ratio between the value of end-diastolic right ventricular area (RVA d) minus end-systolic right ventricular area (RVA s) and end-diastolic area. The rats in control group had no tricuspid regurgitation, we directly assessed the right ventricular end-systolic pressure using right ventricular catheterization technique. The monitoring data was collected and analyzed using LabChart 8 (ADInstruments).

### Quantitative mass spectrometry

According to the data measured by echocardiography, right ventricular tissue (male, 6–8 weeks old, n = 3) were collected at day 28 from euthanized rats and shipped to Jingjie PTM-Biolab for 4D label-free quantitative proteomic analysis. Samples were subjected to standard protein extraction and trypsin digestion [21]. The tryptic peptides were desalted by C18 SPE column and were subsequently dissolved in solvent A (0.1% formic acid, 2% acetonitrile), directly loaded onto a home-made reversed-phase analytical column (25-cm length, 75/100 μm i.d.). Peptides were separated with a gradient from 6 to 24% solvent B (0.1% formic acid in acetonitrile) over 70 min, 24% to 35% in 14 min and climbing to 80% in 3 min then holding at 80% for the last 3 min, all at a constant flow rate of 450 nL/min on a nanoElute UHPLC system (Bruker Daltonics). The peptides were subjected to capillary source followed by the timsTOF Pro (Bruker Daltonics) mass spectrometry. Based on the Raw file obtained from mass spectrometry detection, a database of sample-specific proteins based on the origin was constructed for database search. Subsequently, quality control and quantitative analysis at the peptide and protein levels were performed on the matched results. Fold change (FC) and t-test were performed on the quantitative results. The false discovery rate (FDR) adjusted *p* value was calculated based on the Benjamini and Hochberg (BH) method [22]. Proteins with fold change (FC) above 1.5 or below 1/1.5 having more than one unique peptide were regarded to be differentially expressed (FDR-adjusted p value < 0.05).

### TEPP-46 treatment

The PAH rats were randomly divided into two groups on the 21st day after MCT injection. The experimental group was given TEPP-46 (30 mg/kg; MedChem Express, HY-18657) gavage treatment every day, and the control group was given corresponding solvent gavage operation. Cardiac function data were obtained every 2 days, and right ventricular tissues were harvested on day 28.

### Isolation, culture, and treatment of primary cardiac myocytes

Neonatal rat ventricular myocytes (NRVMs) were isolated from 1-day-old SD rats by using a neonatal heart dissociation kit (Miltenyi Biotec, 130-098-373) following the manufacturer’s instructions and cultured in dulbecco's modified eagle medium (DMEM) (Gibco, 11965092) containing 10% fetal bovine serum (FBS) (Hyclone, SV30087.03), and 1% penicillin & streptomycin (Abiowell, AWH0529a). The cells were grown at 37 °C in a humidified atmosphere with 5% CO_2_, and the medium was changed every two days. primary cardiac myocytes were exposed to arginine vasopressin (AVP, 1 µM, HY-P0049; MedChem Express) for up to 48 h to induce hypertrophy and TEPP-46 (100 µM) for 24 h to promote PKM2 tetramerization.

### Western blotting analysis

Total protein was extracted from right ventricular tissues or myocytes with RIPA buffer (NCM, WB2100) containing 1 mM PMSF (Byotime, ST507), and protein concentration was determined by BCA protein assay kit (NCM, WB6501). Total proteins were separated on BeyoGel Plus PAGE Tris-Gly system (Byotime, 0469S) and transferred to a polyvinylidene fluoride (PVDF) membrane (Millipore, ISEQ00010). The membrane was blocked with 5% skim milk in PBS with 0.1% Tween 20 (PBST) at room temperature for 1 h and incubated with primary antibodies (Additional file 3: Table S1) at 4 °C overnight. The membrane was then incubated with peroxidase affiniPure goat anti-rabbit IgG (H + L) (1:10000, Jackson, 111-035-144) at room temperature for 1 h. Finally, enhanced chemiluminescence (ECL) reagent (Advansta, K-12045-D50) was used to detect the bands. All images of uncropped gels from figures could be reviewed in Additional file 6.

### Crosslinking to determine PKM2 tetramer

As descirbed in previous studies [23, 24], an equal amount of fresh heart right ventricular tissue or primary myocytes was used for crosslinking. Myocardial tissue was cut into 1 mm^3^ pieces with surgical scissors, then it was crosslinked with 2.5 mM DSS in PBS for 30 min at room temperature, with constant rotation. For primary myocytes, we used 500 μM DSS in PBS to crosslink. Samples were separated by BeyoGel Plus PAGE 4–20% Tris-Gly Gel (Beyotime, P0469S).

### Quantitative real-time PCR

The total RNA of ventricular tissue was isolated using the Total RNA Kit II (R6934-01, Omega Bio-tek) according to the manufacturer’s instructions. An RT Reagent Kit with gDNA Eraser (RR047A, Takara) was used for cDNA synthesis. Real-time PCR was performed using All-in-OneTM qPCR Mix (QP001, GeneCopoeia) according to the manufacturer’s protocol. Primer sequences are listed in Additional file 4: Table S2.

### Histology and immunochemistry

Paraffin-embedded sections were subjected to hematoxylin and eosin (HE) or Masson’s trichrome staining. Images were acquired using a Nikon Eclipse E100 and a NIKON DS-U3 automated slide scanner.

### TUNEL staining

The heart samples were separated and fixed in 10% phosphate-buffered formalin for 24 h, subsequently embedded in paraffin, and sliced (4–5 μm). Terminal deoxynucleotidyl transferase-mediated dexoxyuridine triphosphate nick-end labeling (TUNEL) staining was performed using the TUNEL BrightGreen Apoptosis Detection Kit (A112-01, Roche Life Science) following manufacturer’s instructions. Apoptotic nuclei were labeled with green fluorescein staining and total cardiomyocyte nuclei were marked with 4′,6′-diamidino-2-phenylindole (DAPI) (SouthernBiotech, 0100-20). The slices of heart tissues were viewed by confocal microscopy (Eclipse C1, NIKON). The rate of apoptosis was displayed as the percentage of TUNEL positive nuclei in DAPI-stained nuclei.

### Flow cytometry analysis

Cells were digested with trypsin without EDTA and washed twice with PBS. Apoptosis of myocytes was detected by PE Annexin V Apoptosis Detection Kit I (BD Biosciences, 559,763) following the manufacturer’s instructions. Flow cytometry analysis was performed on a DxP AthenaTM instrument (Cytekbio), and the data were analysed using FlowJo software. Annexin V with PE staining was monitored at emission wavelength of 578 nm and an excitation wavelength of 488 nm. 7-Amino-Actinomycin (7-AAD) was monitored at emission wavelength of 647 nm and an excitation wavelength of 488 nm. Cells negative for Annexin V and 7-AAD staining were deemed to be viable. Cells in early apoptosis displayed positive for Annexin V while maintaining negative for 7-AAD staining. Conversely, cells in late apoptosis exhibit positive staining for both Annexin V and 7-AAD.

### Transmission electron microscopy (TEM) and evaluation of damaged mitochondria

Fresh heart right ventricle samples were fixed in 2.5% glutaraldehyde (pH 7.4) for 2 h. Samples were then washed three times with 0.1 M phosphate buffer (pH 7.2) and fixed in 1% osmic acid at 4 ℃ for 2 h. Subsequently, the samples were gradient dehydrated in a graded series of ethanol, embedded in Epon-Araldite resin for penetration and placed in a mold for polymerization. After the semi thin section was used for positioning, the ultrathin section was made and collected for microstructure analysis, followed by the counterstaining of 3% uranyl acetate and 2.7% lead citrate. Data were acquired with a Hitach HT7800 transmission electron microscope.

Healthy mitochondria exhibit intact and well-defined membranes, along with abundant and regular cristae. Swollen mitochondria, on the other hand, display distorted membranes and disrupted or enlarged cristae. Vacuolar mitochondria are characterized by the separation of inner and outer mitochondrial membranes, accompanied by the absence of cristae. To quantify damaged mitochondria, the ratio of vacuolar and swollen mitochondria to the total number of mitochondria in the image was computed. Ruptured mitochondria exhibit a discontinuity in the outer mitochondrial membrane. To quantify ruptured mitochondria, the ratio of ruptured mitochondria to the total number of mitochondria in the image was calculated.

### Advanced oxidation protein product (AOPP) measurement

AOPP test was used to assess oxidative stress within the right ventricular tissues according to the manufacturer’s instructions. Right ventricular myocardial tissue samples (20–30 mg) were harvest for homogenization. Then, we collected the supernatant for AOPP measurements using the AOPP assay kit (Abcam, ab242295). Results were normalized by protein concentration.

### ROS measurement in primary cardiac myocytes

Cells were seeded in 35 mm glass-bottomed culture dish with a density of 5 × 10^4^/ml. Cells were incubated with 5 μM MitoSOX Red (Invitrogen, M36008) for 10 min at 37 ℃. The cells were washed twice with PBS, and the fluorescence imaging of live primary cardiomyocyte was conducted at 510/580 nm on Axio Observer 7 microscope (Zeiss). The mean fluorescence intensity of cells was quantified using Fiji software.

### Immunofluorescence

Cells were stained with 100 nM MitoTracker Red (Invitrogen, M7512) for 30 min, fixed for 10 min with 4% paraformaldehyde and permeabilized with 0.1% Triton X-100 for 10 min. For OPA1 staining, cells were incubated with specific primary antibody (CST, 67589S) overnight in 1% BSA at 4 °C, followed by secondary antibody (Invitrogen, A11034) at room temperature for 1 h. DNA was stained with DAPI. Images were acquired using Apotome microscope (Zeiss). As previously described, the means of area, perimeter, aspect ratio and form factor of mitochondria were quantified using Mitochondria Analyzer in Fiji software according to the published protocol [25–27].

### Statistical Analysis

All replicate data are expressed as the means ± standard error of the mean (SEM). The distribution of the data was assessed with the Shapiro–Wilk normality test. The significance of difference between two independent experimental groups was assessed using unpaired Welch’s t-test, and the significance of difference among more than two groups was assessed using one-way ANOVA. The data between two groups with repeated measurements were compared using two-way ANOVA. The value of statistical significance was set at *p* < 0.05. *, ** and *** represents *p* < 0.05, < 0.01, and < 0.001, respectively. Statistical analyses were performed using GraphPad Prism 9 software.

## Results

### Oxidative stress and mitochondrial disorganization are associated with increased apoptosis in the right ventricular tissue of RVF rats

**Fig. 1:**
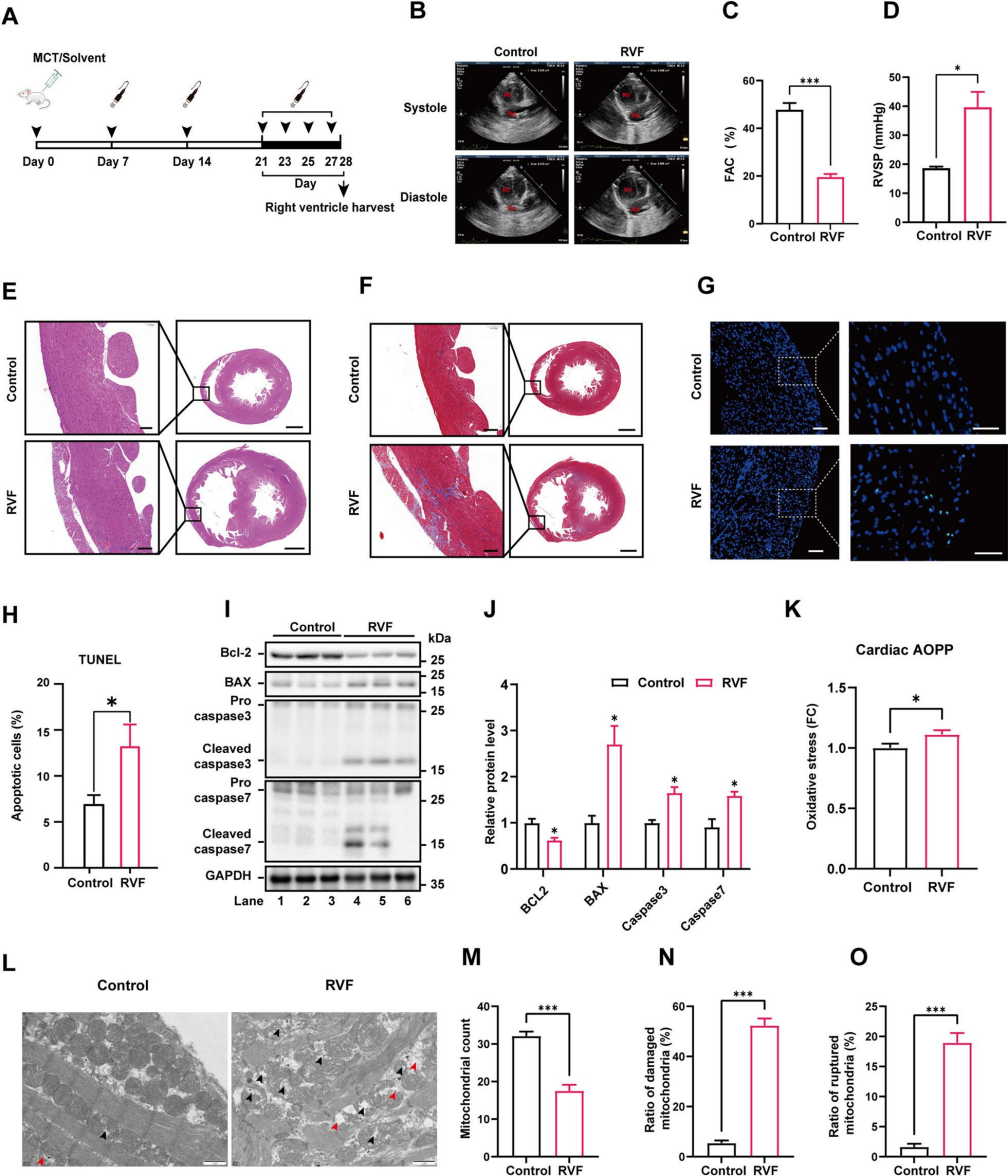
Oxidative stress and mitochondrial disorganization are associated with increased apoptosis in the right ventricular tissue of RVF rats. **A** Schematic diagram of the pulmonary hypertension-induced RVF rat model established by intraperitoneal injection of MCT. Transthoracic echocardiography was carried out as indicated. The right ventricular tissues were harvested on day 28 for analysis. **B**–**C** Right ventricular function was determined by measuring the fractional area change (FAC). Results are expressed as mean ± SEM, n = 6 rats per group. **D** Pulmonary artery pressure was measured using right heart catheterization. Results are expressed as mean ± SEM, n = 4–5 rats per group. **E–F** Paraffin-embedded sections of heart tissue were subjected to HE or Masson staining. The boxed area was further enlarged. Scale bar (left panel) = 100 μm; scale bar (right panel) = 2000 μm. **G** Apoptosis was measured by TUNEL staining (green) at paraffin-embedded sections. Nuclei were stained with DAPI in blue. Scale bar (left panel) = 100 μm; scale bar (right panel) = 50 μm. **H** The quantification of G. Data was collected from ten random fields from the right ventricular tissue section of two rats per group. **I–J** Lysates from right ventricle of control and RVF rats were analyzed by western blotting with indicated antibodies. Relative protein level was quantified and expressed as mean ± SEM, n = 3 rats per group. **K** Relative ROS level in right ventricular tissue was measured by AOPP test. Results are expressed as mean ± SEM, n = 6–7 rats per group. **L** TEM of right ventricle from control and RVF rats (scale bar = 1 μm). Damaged mitochondria were marked with black arrow head, ruptured mitochondria were marked with red arrow head.** M** The total count of mitochondria in field were calculated according to representative transmission electron micrographs of right ventricular tissues of rat in control and RVF group. Results are expressed as mean ± SEM, n = 10 micrographs per group. **N** The ratio of damaged mitochondria to total mitochondria (black arrows) in field were calculated according to representative transmission electron micrographs of right ventricular tissues of rat in control and RVF group. Results are expressed as mean ± SEM, n = 10 micrographs per group. **O** The ratio of ruptured mitochondria to total mitochondria (red arrows) in field were calculated according to representative transmission electron micrographs of right ventricular tissues of rat in control and RVF group. Results are expressed as mean ± SEM, n = 10 micrographs per group

To establish the animal model of RVF, we induced PAH in rats by intraperitoneal injection of MCT as in previous studies [28, 29] (Fig. 1A). The elevation of pulmonary artery pressure was confirmed through transthoracic ultrasound examination and right heart catheterization (Additional file 1: Fig. S1A, B). RVF rats exhibited tricuspid regurgitation on ultrasound imaging starting from day 21 after MCT injection (Additional file 1: Fig. S1C), accompanied by a rapid deterioration in cardiac function (Fig. 1B–D). HE and Masson staining showed severe loss of cardiomyocytes and accumulation of fibrous components in the right heart tissue of RVF rats comparing to the control group (Fig. 1E, F). Apoptosis was significantly activated in the right ventricular tissue of RVF rats (Fig. 1G, H). Correspondingly, the expression of proapoptotic proteins (BAX, caspase-3 and caspase-7) was increased, while the level of anti-apoptotic protein Bcl-2 was downregulated (Fig. 1I, J). Meanwhile, the oxidative stress level was significantly upregulated in the right ventricular tissue of RVF rats, accompanied by the disorganized mitochondria with reduced density, significantly increased swelling, vacuolization and rupture of outer membrane (Fig. 1K–O).

### PKM2 is upregulated during right ventricular failure and negatively correlated with the cardiac function

**Fig. 2:**
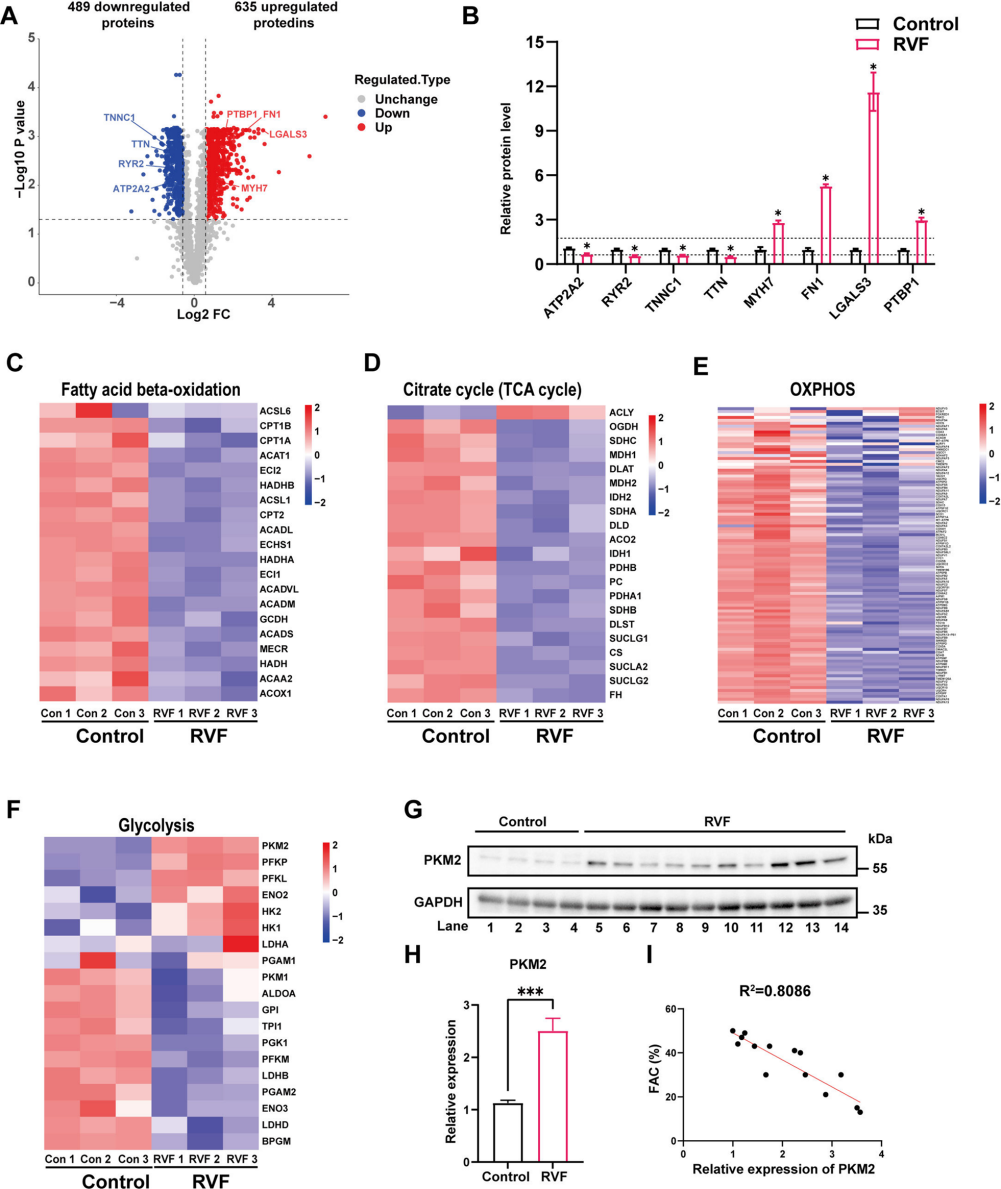
Quantitative proteomics reveals that PKM2 is upregulated during right ventricular failure and negatively correlated with the cardiac function**.** **A** The volcano plot of proteins quantified by mass spectrometry from right ventricular tissue of control and RVF rats. **B** Relative abundance of proteins known to be involved in heart failure from mass spectrometry. The dash lines represent the upregulation and downregulation thresholds. **C–F** Heatmaps of differentially expressed proteins related to fatty acid beta-oxidation, TCA cycle, OXPHOS and glycolysis from mass spectrometry. **G–H** Lysates from right ventricle of control and RVF rats were analyzed by western blotting with indicated antibodies. Relative protein level was quantified and expressed as mean ± SEM, n = 4 rats in control group, n = 10 in RVF group. **I** The relative protein level of PKM2 was negatively correlated with the right ventricular function

To explore the molecular mechanisms regulating heart failure progression, the right ventricular tissues of RVF rats and control group were subjected to 4D label-free quantitative liquid chromatography-mass spectrometry analysis. Principal component analysis (PCA) showed distinct proteomic profile between RVF rats and control (Additional file 1: Figure S1D). Proteins with FC above 1.5 or below its reciprocal having more than one unique peptide were regarded to be differentially expressed (FDR-adjusted *p* value < 0.05). Among the 2253 identified proteins, 635 were upregulated and 489 were downregulated comparing RVF rats and control group (Fig. 2A, Additional file 5: Table S3). As expected, proteins known to be involved in heart failure were enriched. The proteins regulating calcium ion cycle and contraction unit (ATP2A2, RYR2, TNNC1 and TTN) were significantly downregulated, while the proteins involved in cardiomyocyte hypertrophy, fibrosis and transcription reprogramming (MYH7, FN1, LGALS3 and PTBP1) were significantly upregulated (Fig. 2B) [30–35]. Moreover, the expression level of genes involved in myocardial contractile function was analyzed by RT-qPCR. The expression of Atp2a2, Ryr2, Myh6, Tnnt3 and Tnni3 was significantly downregulated in RVF rats, indicating the myocardial contractility was severely impaired (Additional file 1: Fig. S1E). Importantly, most proteins participated in fatty acid oxidation, tricarboxylic acid cycle (TCA) cycle and oxidative phosphorylation (OXPHOS) were significantly downregulated (Fig. 2C–E), indicating a reprogramming of energy metabolism away from mitochondrial respiration in the right ventricular tissue of RVF rats [14]. Indeed, key enzymes of glycolysis including PKM2 (but not PKM1) were significantly upregulated (Fig. 2F–H, Additional file 1: S1F, G). The PKM2 protein level was negatively correlated with the right ventricular function (Fig. 2I), indicating an important role of PKM2 during heart failure progression.

### Promoting PKM2 tetramerization alleviates oxidative stress and apoptosis during cardiomyocyte hypertrophy

**Fig. 3:**
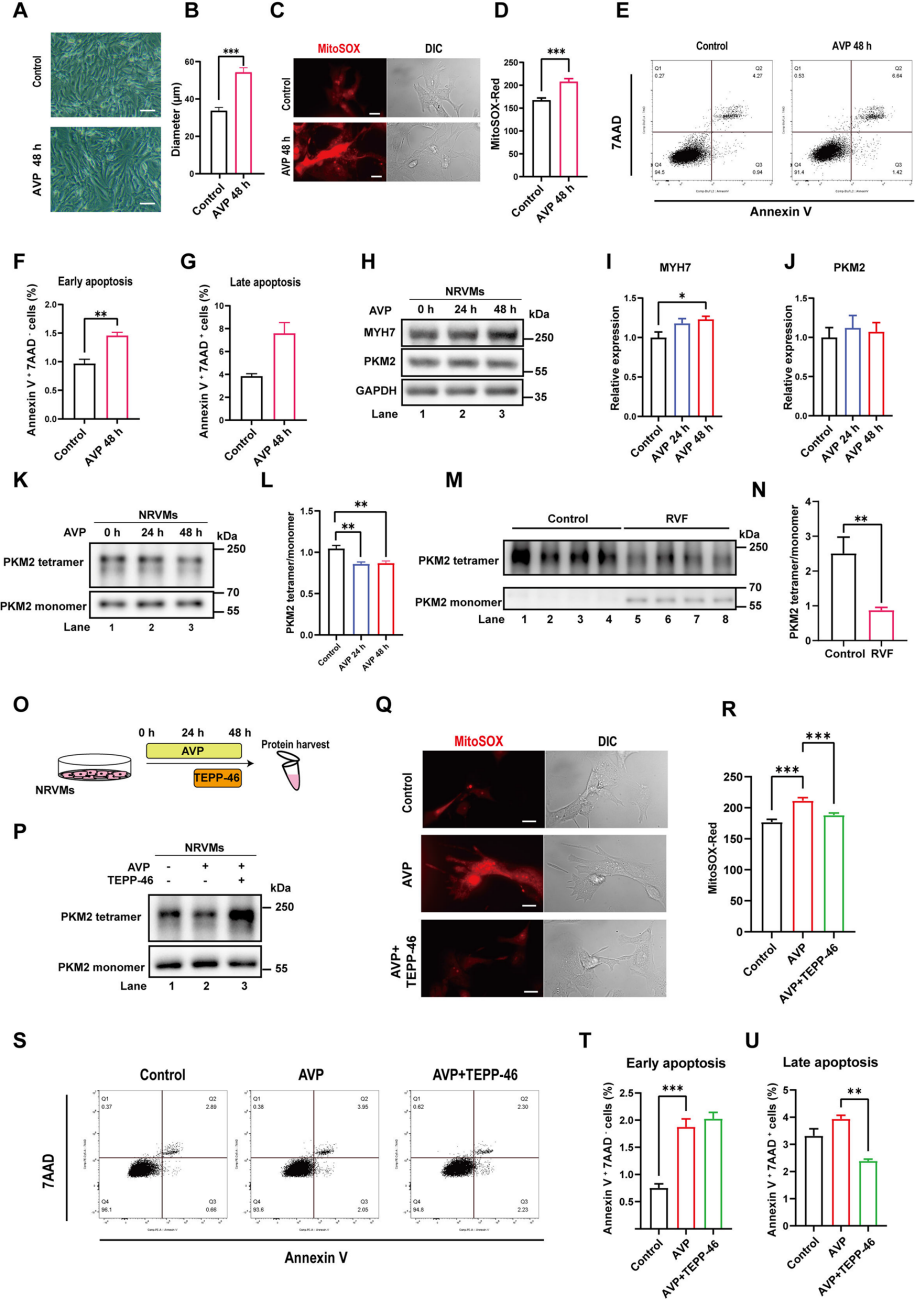
Promoting PKM2 tetramerization alleviates oxidative stress and apoptosis during cardiomyocyte hypertrophy. **A** Rat primary cardiomyocytes were treated with 1 μM AVP for 48 h to induce hypertrophy. Scale bar = 200 μm. **B** The diameter of cardiomyocytes in A was measured. Results are expressed as mean ± SEM, n = 20 cells per group. **C–D** The ROS level was analyzed by MitoSOX Red. Scale bar = 20 μm. Quantitative results are expressed as mean ± SEM, n = 20 cells per group. **E–G** The apoptosis of cardiomyocytes was analyzed by flow cytometry. Quantitative results are expressed as mean ± SEM, n = 3. **H–J** Lysates from primary cardiomyocytes were analyzed by western blotting with indicated antibodies. Relative protein level was quantified and expressed as mean ± SEM, n = 3. **K–L** As in previous studies, PKM2 tetramerization was analyzed by DSS crosslinking of primary cardiomyocytes. The ratio of tetramer to monomer was quantified and expressed as mean ± SEM, n = 3. **M–N** PKM2 tetramerization in the right ventricular tissue of control and RVF rats was analyzed by DSS crosslinking. The ratio of tetramer to monomer was quantified and expressed as mean ± SEM, n = 4 rats per group. **O–P** Rat primary cardiomyocytes were treated with 1 μM AVP for 48 h and 100 μM TEPP-46 for 24 h as indicated. PKM2 tetramerization was analyzed by DSS crosslinking. **Q–R** The ROS level was analyzed by MitoSOX Red. Scale bar = 20 μm. Quantitative results are expressed as mean ± SEM, n = 20 cells per group. **S–U** The apoptosis of cardiomyocytes was analyzed by flow cytometry. Quantitative results are expressed as mean ± SEM, n = 3

Cardiomyocyte hypertrophy is a key phenotype in the progression of heart failure. To further study the molecular mechanism underlying the function of PKM2, we stimulated NRVMs with AVP to induce hypertrophy as in previous studies [36, 37]. Application of AVP effectively induced cardiomyocyte hypertrophy (Fig. 3A, B), oxidative stress (Fig. 3C, D) and apoptosis (Fig. 3E–G). Unlike in the RVF rats, cardiomyocyte hypertrophy induced by AVP did not upregulate the steady level of PKM2, while MYH7 expression was increased (Fig. 3H–J). Interestingly, PKM2 tetramerization was significantly downregulated in cardiomyocyte hypertrophy as well as in the right ventricular tissue of RVF rats (Fig. 3K–N). The destabilization of PKM2 tetramer promotes tumor growth under oxidative stress [8, 10, 13]. To analyze the function of PKM2 tetramerization in cardiomyocyte hypertrophy, rat primary cardiomyocytes were further treated with TEPP-46 following AVP stimulation to promote PKM2 tetramerization (Fig. 3O, P) [24, 38]. Surprisingly, unlike in tumor cells, promoting PKM2 tetramerization alleviated the oxidative stress and accompanied apoptosis during cardiomyocyte hypertrophy (Fig. 3Q–U).

### Facilitating PKM2 tetramerization promoted mitochondrial network formation by regulating mitochondrial fission and fusion machineries

**Fig. 4:**
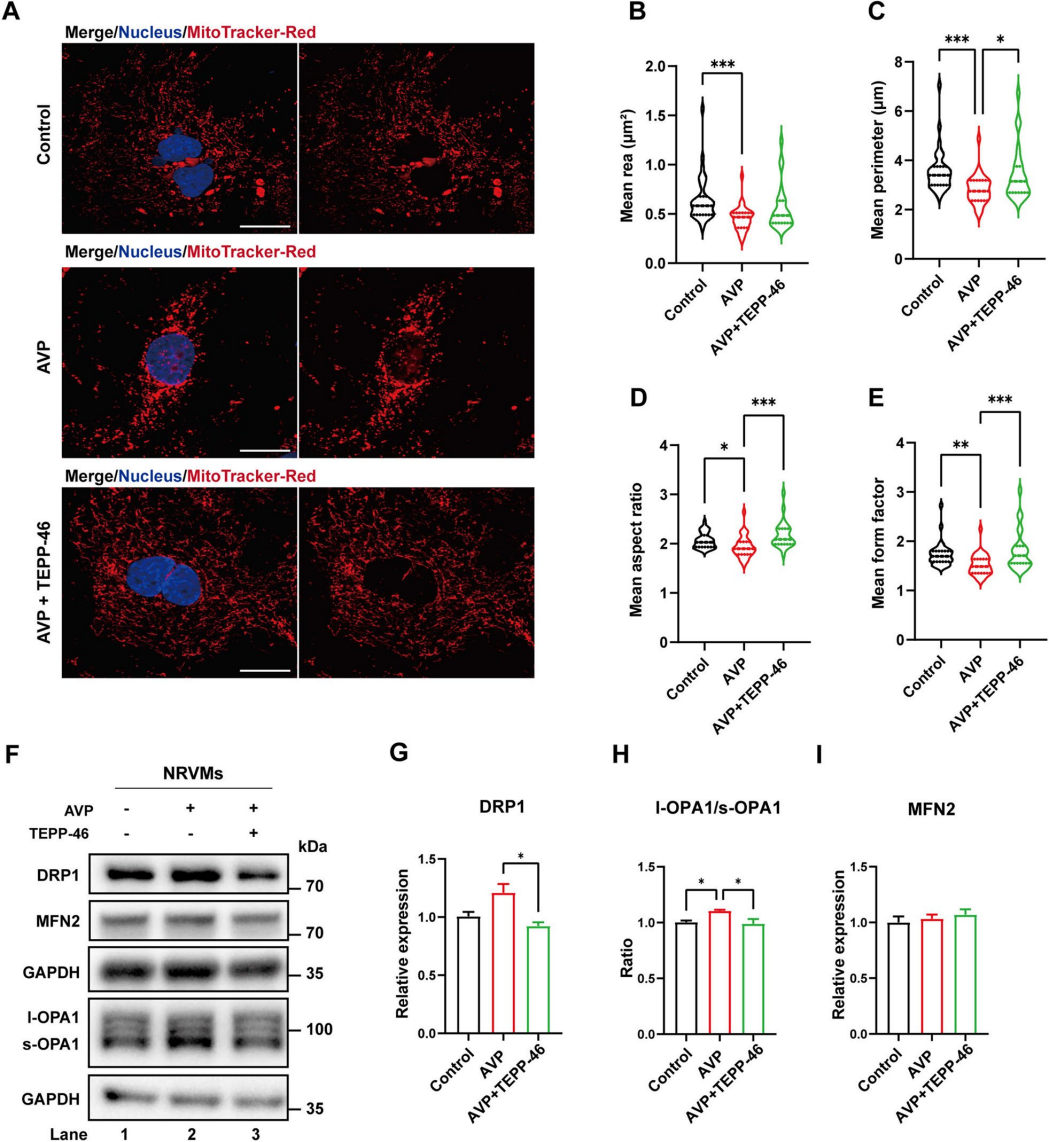
Facilitating PKM2 tetramerization promoted mitochondrial network formation. **A** Rat primary cardiomyocytes were treated with AVP and TEPP-46 as indicated and stained by MitoTracker-Red to visualize mitochondrial morphology. Scale bar = 20 µm. **B-E** The means of area, perimeter, aspect ratio and form factor of mitochondria were quantified. Results are expressed as mean ± SEM, n = 25 cells per group. **F–I** Lysates from primary cardiomyocytes were analyzed by western blotting with indicated antibodies. Relative protein level was quantified and expressed as mean ± SEM, n = 3

Oxidative stress is intimately connected to mitochondrial morphology. We thus analyzed AVP treated cardiomyocytes via immunofluorescence and stained for mitochondrial specific markers (mitochondrial protein OPA1 and MitoTracker Red). Mitochondrial network was found to be more fragmented in hypertrophic cardiomyocytes (Additional file 2: Fig. S2A). Mean mitochondrial area and mean mitochondrial perimeter in hypertrophic cardiomyocytes were significantly lower than those in control cells (Additional file 2: Fig. S2B, C), and the decrease in mean form factor and mean aspect ratio suggested that the mitochondria in hypertrophic cardiomyocytes were more spherical in shape, indicating increased mitochondrial fission in cardiomyocyte hypertrophy (Additional file 2: Fig. S2D, E). Accordingly, the expression of Drp1 and cleaved OPA1 was elevated in AVP treated cardiomyocytes, which was consistent with the increased mitochondrial fission in hypertrophic cardiomyocytes (Additional file 2: Fig. S2F–I). To analyze how PKM2 tetramerization regulates mitochondrial dynamics in hypertrophic cardiomyocytes, PKM2 tetramerization was promoted by TEPP-46 following AVP stimulation. Importantly, enhanced PKM2 tetramerization suppressed mitochondrial fission in hypertrophic cardiomyocytes (Fig. 4A–E). Meanwhile, western blotting analysis demonstrated the increased expression of Drp1 and the elevated cleavage of OPA1 were both rescued by TEPP-46 treatment (Fig. 4F–I). Taken together, facilitating PKM2 tetramerization promoted mitochondrial network formation by regulating mitochondrial fission and fusion machineries.

### Enhancing PKM2 tetramer formation improves cardiac mitochondrial morphology, mitigates oxidative stress and alleviates heart failure

**Fig. 5:**
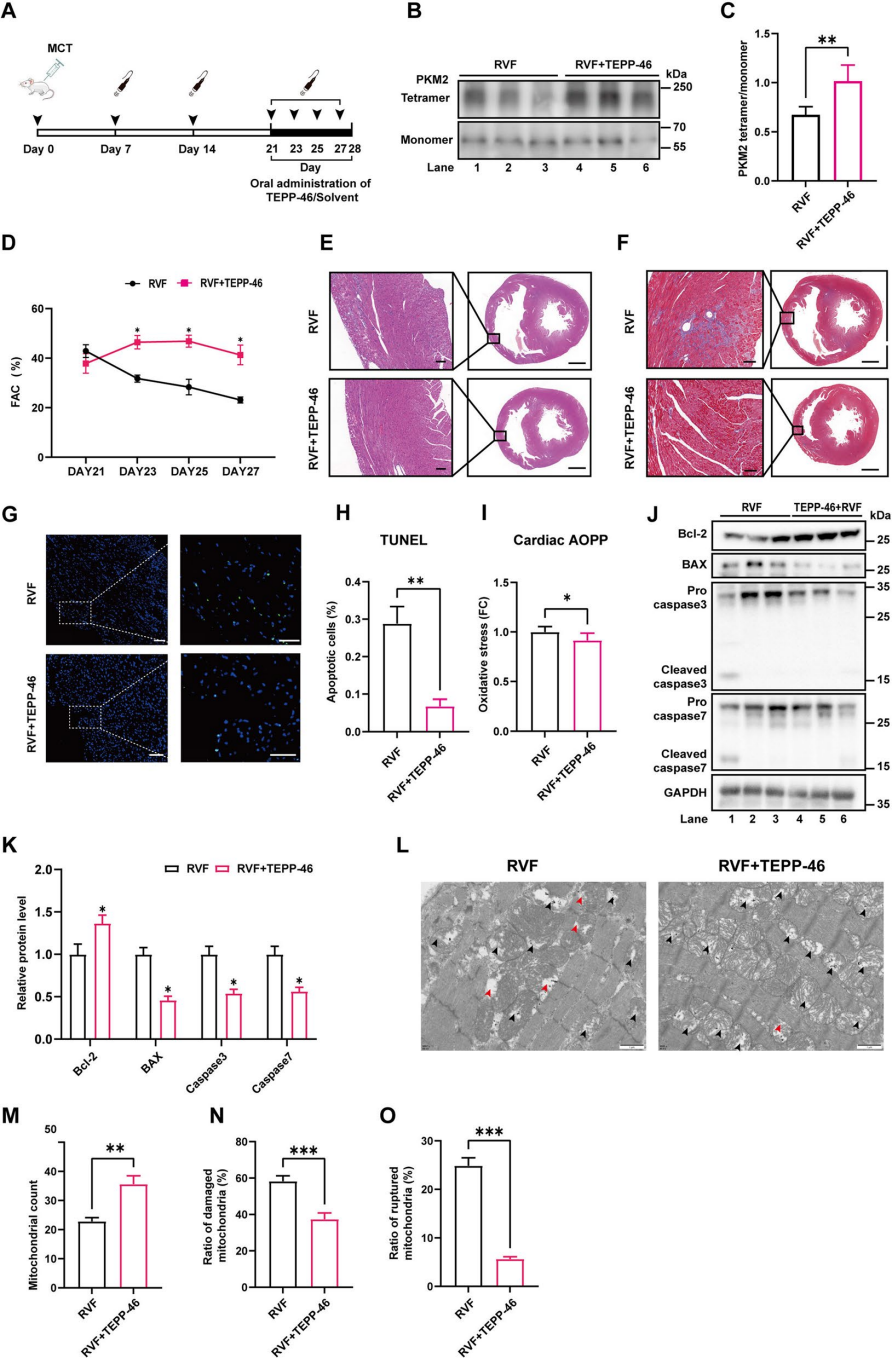
Oral administration of TEPP-46 alleviates heart failure, improves cardiac mitochondrial morphology and mitigates oxidative stress. **A** Schematic diagram of the TEPP-46 treatment for RVF rats. Transthoracic echocardiography was carried out as indicated. The right ventricular tissues were harvested on day 28 for analysis. **B–C** PKM2 tetramerization in the right ventricular tissue of RVF and RVF + TEPP-46 rats were analyzed by DSS crosslinking. The ratio of tetramer to monomer was quantified and expressed as mean ± SEM, n = 5 rats per group. **D** Right ventricular function of RVF rats treated with or without TEPP-46 was determined by measuring the FAC at indicated time. Significance was determined by two-way ANOVA followed by Sidak's multiple comparisons test, n = 5 rats per group. **E–F** Paraffin-embedded sections of heart tissue were subjected to HE or Masson staining. The boxed area was further enlarged. Scale bar (left panel) = 100 μm; scale bar (right panel) = 2000 μm. **G** Apoptosis was measured by TUNEL staining (green) at paraffin-embedded sections. Nuclei were stained with DAPI in blue. Scale bar (left panel) = 100 μm; scale bar (right panel) = 50 μm. **H** The quantification of F. Data was collected from ten random fields of the right ventricular tissue section of two rats per group. **I** Relative ROS level in right ventricular tissue was measured by AOPP test. Results are expressed as mean ± SEM, n = 6 rats per group. **J–K** Lysates from right ventricle of RVF rats treated with or without TEPP-46 were analyzed by western blotting with indicated antibodies. Relative protein level was quantified and expressed as mean ± SEM, n = 3 rats per group. **L** TEM of right ventricle in RVF and RVF + TEPP-46 rats (Scale bar = 1 µm). Damaged mitochondria were marked with black arrow head, ruptured mitochondria were marked with red arrow head.** M** The total count of mitochondria in field were calculated according to representative transmission electron micrographs of right ventricular tissues of rat in RVF and RVF + TEPP-46 group. Results are expressed as mean ± SEM, n = 10 micrographs per group. **N** The ratio of damaged mitochondria to total mitochondria (black arrows) in field were calculated according to representative transmission electron micrographs of right ventricular tissues of rat in RVF and RVF + TEPP-46 group. Results are expressed as mean ± SEM, n = 10 micrographs per group. **O** The ratio of ruptured mitochondria to total mitochondria (red arrows) in field were calculated according to representative transmission electron micrographs of right ventricular tissues of rat in RVF and RVF + TEPP-46 group. Results are expressed as mean ± SEM, n = 10 micrographs per group

**Fig. 6:**
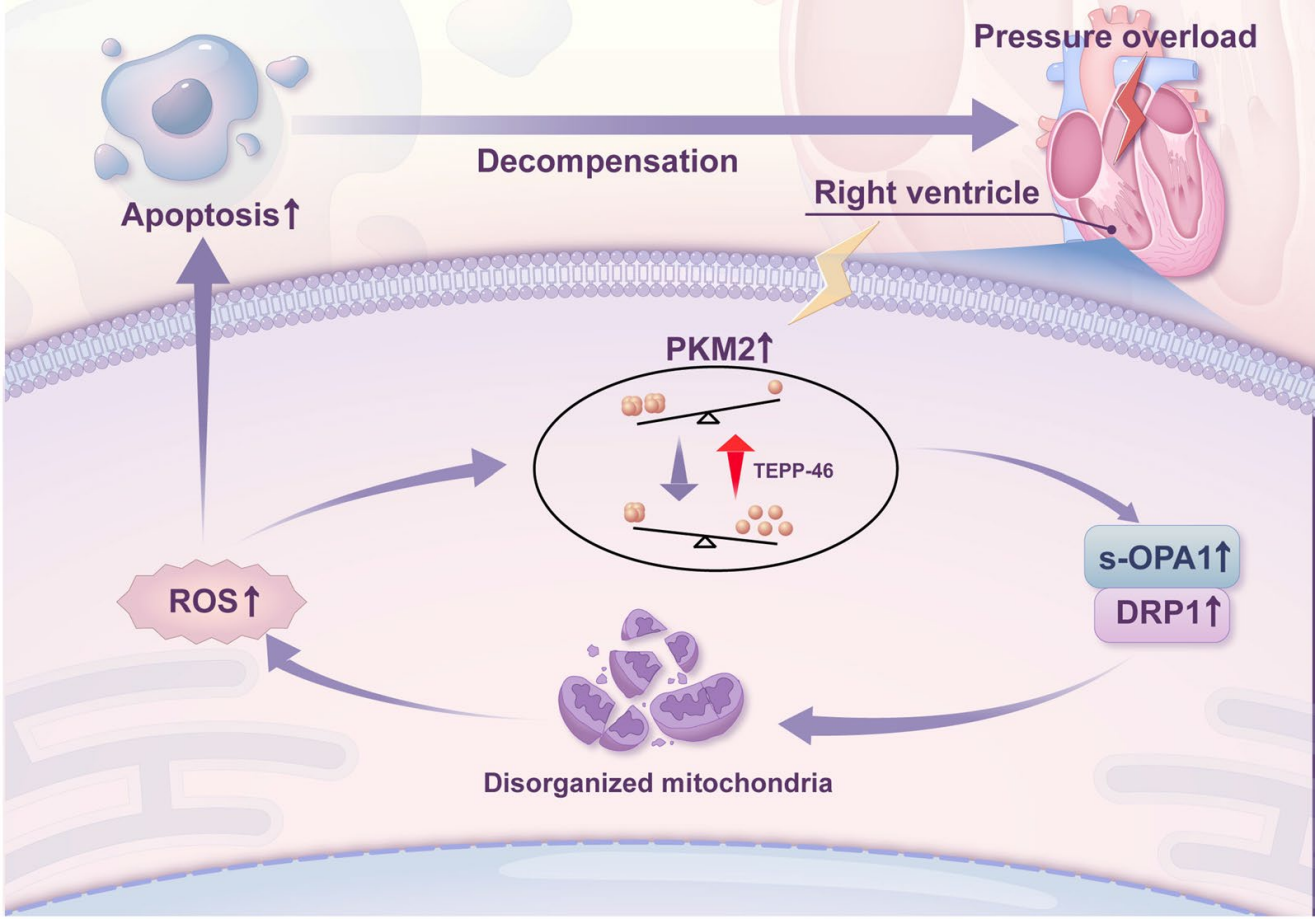
Schematic model of promoting PKM2 tetramerization alleviates the decompensation of cardiac function by regulating oxidative stress and mitochondrial dynamics. Pressure overloading induces cardiomyocyte hypertrophy with upregulated PKM2 level, and the tetrameric proportion of PKM2 is reduced by intracellular oxidative stress. An imbalanced ratio of PKM2 tetramer/monomer upregulates Drp1 level and increases OPA1 cleavage, and ultimately leads to mitochondria disorganization and elevated ROS level. This vicious cycle eventually accelerates the apoptosis of cardiomyocytes and promotes cardiac function into the decompensated phase. The application of TEPP-46 can improve mitochondrial network formation, mitigates oxidative stress and alleviates the decompensation of cardiac function via enhancing PKM2 tetramer formation

To investigate whether the deterioration of cardiac function in RVF rats could be alleviated by promoting PKM2 tetramerization, TEPP-46 was administered (30 mg/kg, once per day) by gavage from day 21 to day 27 after intraperitoneal injection of MCT, while the control RVF rats were given the corresponding solvent (Fig. 5A). The right ventricular function was measured every 2 days from day 21. Oral administration of TEPP-46 promoted PKM2 tetramerization in the right ventricular tissue of RVF rats (Fig. 5B, C), and significantly delayed the deterioration of cardiac function without affecting the pulmonary artery pressure (Fig. 5D, Additional file 1: S1H, I). The cardiomyocyte loss and the accumulation of fibrous components in the right ventricular tissue of RVF rats were alleviated via TEPP-46 administration (Fig. 5E, F). Accordingly, TUNEL staining revealed that the apoptosis of cardiomyocyte was decreased with TEPP-46 treatment (Fig. 5G, H), with lower level of oxidative stress in the right ventricular tissue (Fig. 5I). Meanwhile, the dysregulation of anti-apoptotic and proapoptotic proteins in the right ventricular tissue of RVF rats was partially rescued after TEPP-46 administration (Fig. 5J, K). Moreover, the morphological abnormality of mitochondria was largely rescued by promoting PKM2 tetramer formation (Fig. 5L–O). Taken together, enhancing the tetramerization of PKM2 improved mitochondrial morphology, mitigated the oxidative stress related cardiomyocyte apoptosis, and alleviated the deterioration of cardiac function during heart failure progression (Fig. 6).

## Discussion

Right heart failure is primarily caused by pulmonary arterial hypertension and is the leading cause of mortality in affected patients [39]. The increased afterload, along with various endocrine hormones, promotes hypertrophy of myocardial cells. However, hypertrophied myocardial cells eventually progress towards apoptosis, leading to the decompensation of heart function [40–42]. Mitochondrial damage and excessive accumulation of ROS are known to promote myocardial cell apoptosis, which is a key event in the decompensation of heart function [15, 43, 44]. Therefore, proper regulation of mitochondrial quality control and oxidative stress in myocardial cells may be important aspects for preserving heart health and delaying the progression of heart failure. In recent years, numerous studies have reported that the upregulation of PKM2 in the pathological state of the heart plays crucial roles in the occurrence and progression of various types of heart failure [8, 17–19, 44–46]. However, the associated molecular mechanisms are not completely understood.

MCT serves as a classic animal model for pulmonary hypertension. The elevated RVSP induced by MCT in our study align with previous reported findings [28, 29]. Via quantitative proteomics, we observed that the expression of PKM2 was upregulated in the progression of RVF and was negatively correlated with the cardiac function. Interestingly, the tetramerization of PKM2 was significantly downregulated both in cardiomyocyte hypertrophy and in the right ventricular tissue of RVF rats. In tumor cells, the destabilization of PKM2 tetramer restricts its catalytic activity in glycolysis, diverts glucose carbon into the pentose phosphate pathway for NADPH production, thus is important for the adaptive response of tumor cells against oxidative stress to promote tumor cell proliferation and tumor growth [8, 48]. However, the adaptation of oxidative stress in cardiomyocyte seems to be differently regulated. Unlike in tumor cells, we observed that facilitating PKM2 tetramerization in cardiomyocytes alleviated oxidative stress. The exact molecular mechanisms behind this phenomenon are not clear, but the function of mitochondrial dynamic control is worthy of further studies, since stabilization of PKM2 tetramer improved mitochondrial network formation and mitigated apoptosis and heart failure.

Proper mitochondrial dynamic control is crucial for healthy cardiac function [48]. The abnormal regulation of mitochondrial fusion/fission machineries is intimately associated with mitochondrial dysfunction during heart failure [49–53]. In our study, the increased mitochondrial fragmentation in hypertrophic cardiomyocytes was accompanied by the elevated level of DRP1 expression and OPA1 cleavage, which were effectively rescued by promoting PKM2 tetramerization with TEPP-46. Although we did not observe any alteration on MFN2 by TEPP-46, it does not rule out the impact of PKM2 tetramerization on the fusion machineries of mitochondrial outer membrane. Indeed, in tumor cells, it has been reported that PKM2 could translocate to mitochondria and interact with MFN2 to promote mitochondrial fusion and energy production [54]. In addition, PKM2 has also been reported to stabilize Bcl-2, thus inhibiting apoptosis [55]. However, how the oligomerization status of PKM2 is implicated in these processes, especially in cardiomyocytes, awaits further studies.

TEPP-46, a specific PKM2 tetramerization promoter, has been widely utilized and validated for its efficacy in numerous studies [23, 24, 56]. Several studies have indicated that enhancing PKM2 tetramerization is beneficial for cancer treatment [38, 57, 58], effectively prevents the progression of diabetic glomerular pathology [23] or mitigates doxorubicin-induced cardiomyocyte apoptosis [59]. Our research further substantiates that promoting PKM2 tetramerization using TEPP-46 can alleviate the progression of pulmonary arterial hypertension and right heart failure by improving mitochondrial dynamics. However, due to the involvement of dimeric or monomeric forms of PKM2 in regulating many important cellular functions [12], potential side effects of PKM2 tetramerization therapy remains to be a significant challenge. It has been reported that promoting PKM2 tetramerization may exacerbate the progression of bleomycin induced pulmonary fibrosis [60]. Therefore, more experimental and clinical data are needed in the future to support and guide the use of PKM2 tetramerization therapy in disease treatment.

Our research also has some limitations. In vitro, PKM2 expression was not upregulated during the hypertrophy of NRVMs, which contradicts with the data observed in vivo. We believe that this inconsistency could be attributed to the naturally higher expression levels of PKM2 in fetal hearts [61]. NRVMs we utilized were derived from fetal rats, which is a commonly used cell type for cardiac research [62]. However, this did not undermine our experimental conclusions, that the disruption of PKM2 tetramers during myocardial hypertrophy affected the mitochondrial dynamics of cardiomyocytes. Secondly, we did not perform a knockdown of PKM2, for there have been numerous studies on the effects of PKM2 depletion on the cardiac dysfunction [17, 18], and our research was primarily focus on investigating the influence of altered PKM2 tetramerization on mitochondrial homeostasis in cardiomyocytes and on the progression of heart failure.

In summary, our research revealed that promoting PKM2 tetramerization could effectively improve mitochondrial network formation, and alleviate oxidative stress as well as heart failure. Targeting PKM2 tetramerization may be considered as an intervention strategy for patients with both cancer and heart disease comorbidity.

### Supplementary Information

**Additional file 1: Figure S1. A** Tricuspid regurgitation detected by echocardiography was used to represent right ventricular dysfunction and evaluate right ventricular systolic pressure (RVSP). **B **Right ventricular catheterization was used to confirm the pulmonary artery pressure. **C** Kaplan–Meier curve described the percentage of RVF rats free of tricuspid regurgitation. n = 13. **D **Principal component analysis (PCA) of quantitative proteomic data of RVF rats and control. **E** The relative mRNA level of indicated genes was determined by RT-qPCR. Results are expressed as mean ± SEM, n = 6 rats per group. **F–G** Lysates from right ventricle of control and RVF rats were analyzed by western blotting with indicated antibodies. Relative protein level was quantified and expressed as mean ± SEM, n = 4 rats in control group, n = 10 in RVF group. **H **Tricuspid regurgitation detected by echocardiography for evaluation of RVSP. **I** RVSP was measured by echocardiograph and expressed as mean ± SEM, n = 5 rats per group.

**Additional file 2: Figure S2. A **Rat primary cardiomyocytes were treated with 1 μM AVP for 48 h to induce hypertrophy. Mitochondrial morphology was visualized by MitoTracker-Red and OPA1 staining. Scale bar = 20 µm. **B–E** The means of area, perimeter, form factor and aspect ratio of mitochondria were quantified. Results are expressed as mean ± SEM, n = 50 cells per group. **F–I **Lysates from primary cardiomyocytes treated with AVP for indicated time were analyzed by western blotting with indicated antibodies. Relative protein level was quantified and expressed as mean ± SEM, n = 3.

**Additional file 3: Table S1**. Antibody list.

**Additional file 4: Table S2.** Primer sequences.

**Additional file 5: Table S3.** Total differentially expressed protein.

**Additional file 6:** Uncropped gels from figures.

## Data Availability

The data presented in this study are available on request from the corresponding authors.

## Abbreviations

PKM2: M-type pyruvate kinase 2
RVF: Right ventricular failure
SD: Sprague–Dawley
MCT: Monocrotaline
PAH: Pulmonary arterial hypertension
ATP: Adenosine triphosphate
ADP: Adenosine diphosphate
ROS: Reactive oxygen species
FAC: Fractional area change
RVSP: Right ventricular systolic pressure
RAP: Right atrial pressure
TRmax: The maximum velocity of the tricuspid regurgitant
RVA d: End-diastolic right ventricular area
RVA s: End-systolic right ventricular area
FC: Fold Change
TF: T-test
NRVMs: Neonatal rat ventricular myocytes
DMEM: Dulbecco's modified eagle medium
FBS: Fetal Bovine Serum
AVP: Arginine vasopressin
PVDF: Polyvinylidene fluoride
HRP: Horseradish peroxidase
ECL: Enhanced chemiluminescence
RT-qPCR: Quantitative real-time PCR
HE: Hematoxylin and eosin
TUNEL: Terminal deoxynucleotidyl transferase-mediated dexoxyuridine triphosphate nick-end labeling
DAPI: 4′,6′-Diamidino-2-phenylindole
AOPP: Advanced oxidation protein product
TEM: Transmission electron microscopy
SEM: Standard error of the mean
FDR: False discovery rate
BH: Benjamini and Hochberg
PCA: Principal component analysis
TCA: Tricarboxylic acid cycle
OXPHOS: Oxidative phosphorylation
l-OPA1: Long OPA1 forms
s-OPA1: Short OPA1 forms
PK: Pyruvate kinase
Drp1: Dynamin-Related Protein 1
OPA1: OPA1 Mitochondrial Dynamin Like GTPase
MFN2: Mitofusin 2
SERCA2: Sarcoplasmic/Endoplasmic Reticulum Calcium ATPase 2
PLN: Phospholamban
MYH6: Myosin Heavy Chain 6
MYH7: Myosin Heavy Chain 7
RYR2: Ryanodine Receptor 2
TNNT3: Troponin T3
TNNI3: Troponin I3
TNNC1: Troponin C1
BAX: BCL2 Associated X
Bcl-2: Apoptosis Regulator Bcl-2
