# Supplementary figures and images for: M-type pyruvate kinase 2 (PKM2) tetramerization alleviates the progression of right ventricle failure by regulating oxidative stress and mitochondrial dynamics
Source: J Transl Med. 2023 Dec 7;21:888. doi: 10.1186/s12967-023-04780-6 (PMC10702013; doi:10.1186/s12967-023-04780-6)

Supplementary Figure 1

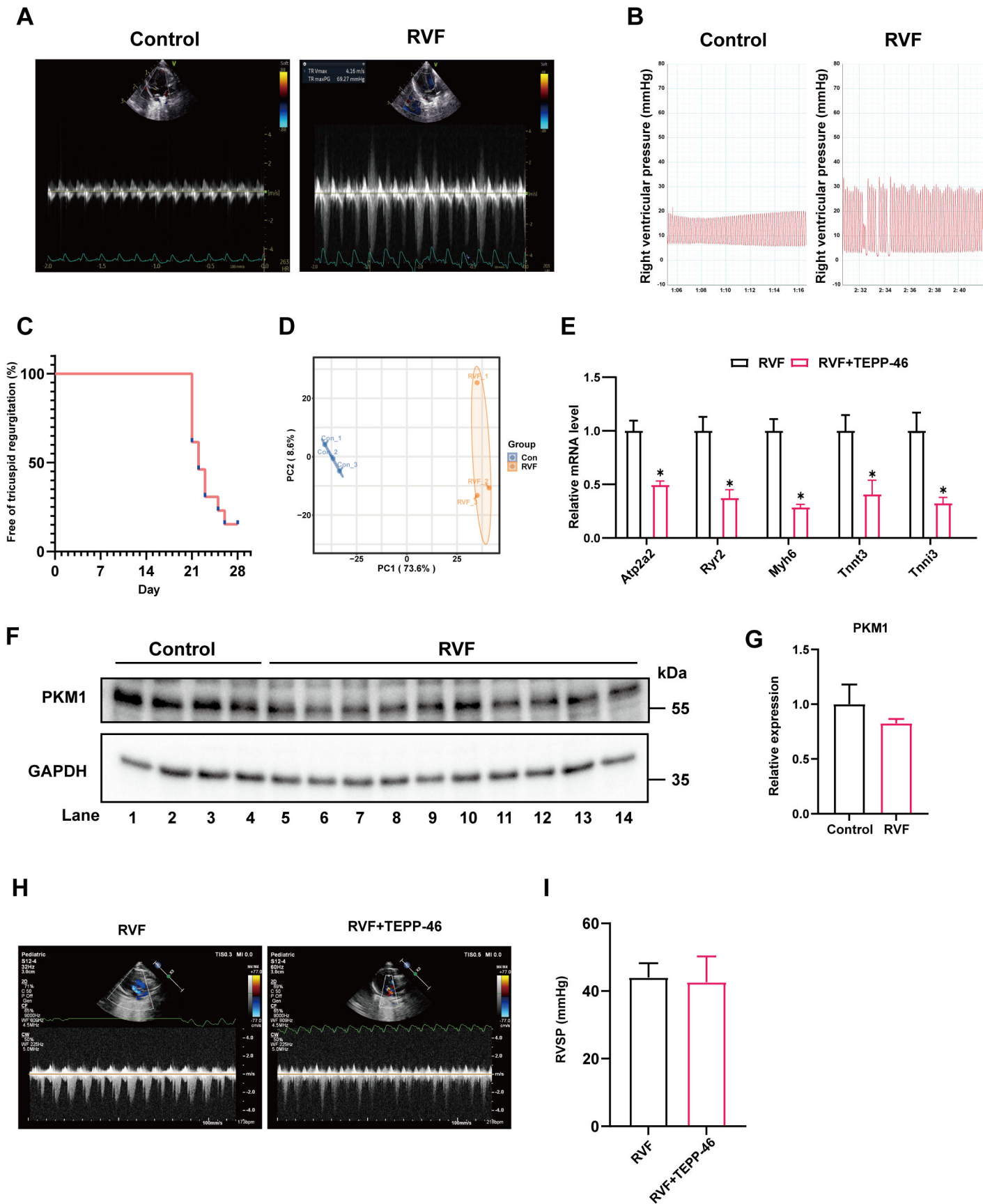

Supplement: Supplementary file 1 — Additional file 1: Figure S1. A Tricuspid regurgitation detected by echocardiography was used to represent right ventricular dysfunction and evaluate right ventricular systolic pressure (RVSP). B Right ventricular catheterization was used to confirm the pulmonary artery pressure. C Kaplan–Meier curve described the percentage of RVF rats free of tricuspid regurgitation. n = 13. D Principal component analysis (PCA) of quantitative proteomic data of RVF rats and control. E The relative mRNA level of indicated genes was determined by RT-qPCR. Results are expressed as mean ± SEM, n = 6 rats per group. F–G Lysates from right ventricle of control and RVF rats were analyzed by western blotting with indicated antibodies. Relative protein level was quantified and expressed as mean ± SEM, n = 4 rats in control group, n = 10 in RVF group. H Tricuspid regurgitation detected by echocardiography for evaluation of RVSP. I RVSP was measured by echocardiograph and expressed as mean ± SEM, n = 5 rats per group. [file 12967_2023_4780_MOESM1_ESM.pdf]

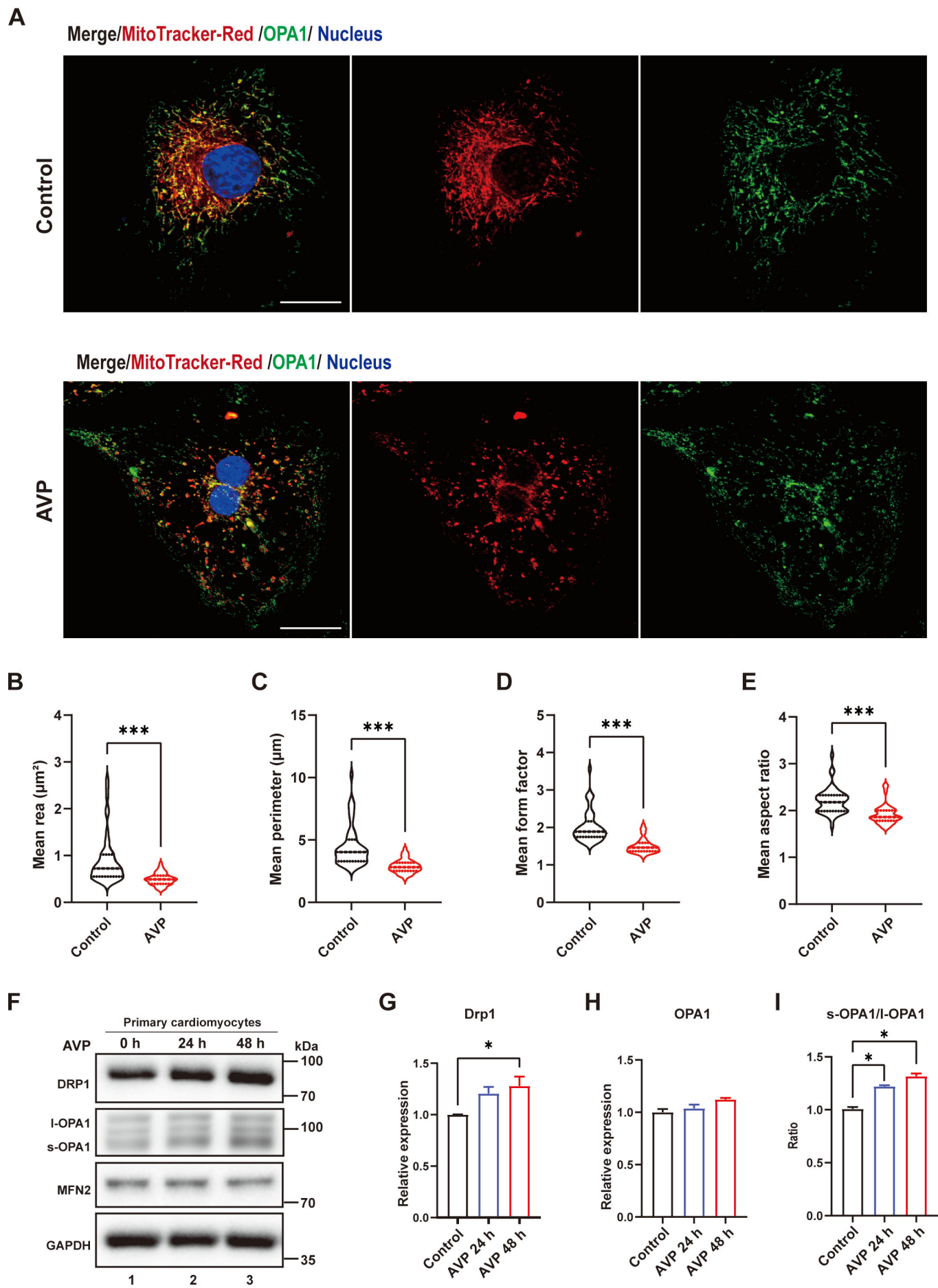

Supplement: Supplementary file 2 — Additional file 2: Figure S2. A Rat primary cardiomyocytes were treated with 1 μM AVP for 48 h to induce hypertrophy. Mitochondrial morphology was visualized by MitoTracker-Red and OPA1 staining. Scale bar = 20 µm. B–E The means of area, perimeter, form factor and aspect ratio of mitochondria were quantified. Results are expressed as mean ± SEM, n = 50 cells per group. F–I Lysates from primary cardiomyocytes treated with AVP for indicated time were analyzed by western blotting with indicated antibodies. Relative protein level was quantified and expressed as mean ± SEM, n = 3. [file 12967_2023_4780_MOESM2_ESM.pdf]
