# Supplementary material for: M-type pyruvate kinase 2 (PKM2) tetramerization alleviates the progression of right ventricle failure by regulating oxidative stress and mitochondrial dynamics
Source: J Transl Med. 2023 Dec 7;21:888. doi: 10.1186/s12967-023-04780-6 (PMC10702013; doi:10.1186/s12967-023-04780-6)
Supplement: Supplementary file 3 — Additional file 3: Table S1. Antibody list. [file 12967_2023_4780_MOESM3_ESM.docx]

**Table S1: Antibody list**

| Protein | Cat No. | Company |
| --- | --- | --- |
| PKM1 | 15821-1-ap | Proteintech |
| PKM2 | 4053S | CST |
| Caspase-3 | ab179517 | Abcam |
| Caspase-7 | ab255818 | Abcam |
| BAX | ab32503 | Abcam |
| Bcl-2 | ab196495 | Abcam |
| OPA1 | 67589S | CST |
| MFN2 | 9482S | CST |
| DRP1 | ab184247 | Abcam |
| GAPDH | YN5585 | Immunoway |
