# Supplementary material for: M-type pyruvate kinase 2 (PKM2) tetramerization alleviates the progression of right ventricle failure by regulating oxidative stress and mitochondrial dynamics
Source: J Transl Med. 2023 Dec 7;21:888. doi: 10.1186/s12967-023-04780-6 (PMC10702013; doi:10.1186/s12967-023-04780-6)
Supplement: Supplementary file 4 — Additional file 4: Table S2. Primer sequences. [file 12967_2023_4780_MOESM4_ESM.docx]

**Table S2: Primer sequences**

| Gene | Forward | Reverse |
| --- | --- | --- |
| Myh6 | TTCCAGAAGCCTCGCAATGTCAAG | CAGCCAGCCCAAGATGTTGTAGTC |
| Tnnt3 | CCAGAGGAGAAACCAAGACCCAAAC | GAGGTCCTTGTTCTGACGCTTCTTC |
| Tnni3 | AAGCAGGTGAAGAAGGAGGACATTG | CAGAGCACAGTGTGAGAGCCATG |
| Tnnc1 | ACGCTGATGGCTACATTGACTTGG | ATCGTTGTTCTTGTCACCGTCCTTC |
| Atp2a2 | AGTTCATCCGCTACCTCATCTCCTC | GCAGACCATCCGTCACCAGATTG |
| Ryr2 | ATCCTAGCCATCCTCCACACCATC | CTCCAACTTCCGTGCCACTTCC |
