# Supplementary material for: M-type pyruvate kinase 2 (PKM2) tetramerization alleviates the progression of right ventricle failure by regulating oxidative stress and mitochondrial dynamics
Source: J Transl Med. 2023 Dec 7;21:888. doi: 10.1186/s12967-023-04780-6 (PMC10702013; doi:10.1186/s12967-023-04780-6)

Figure 1I

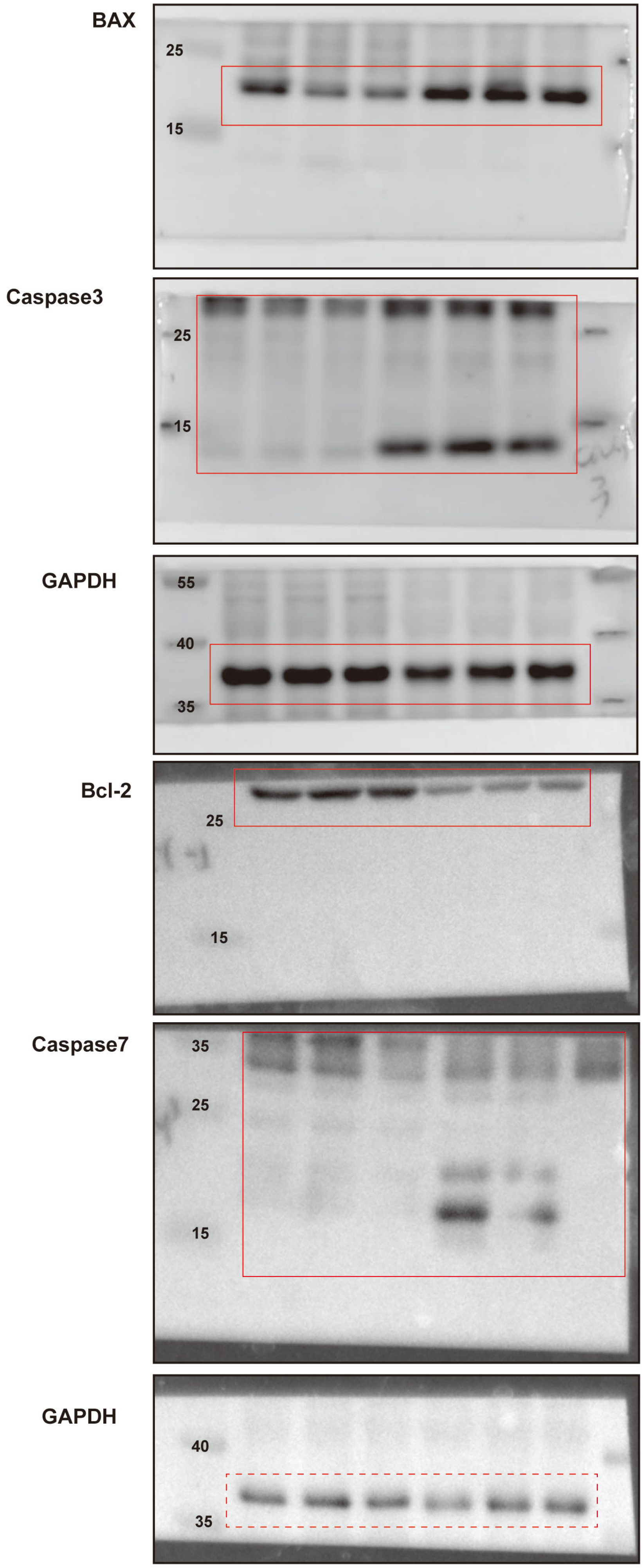

BAX
25
15
Caspase3
25
15
GAPDH
55
40
35
Bcl-2
25
15
Caspase7
35
25
15
GAPDH
40
35

Figure 2G

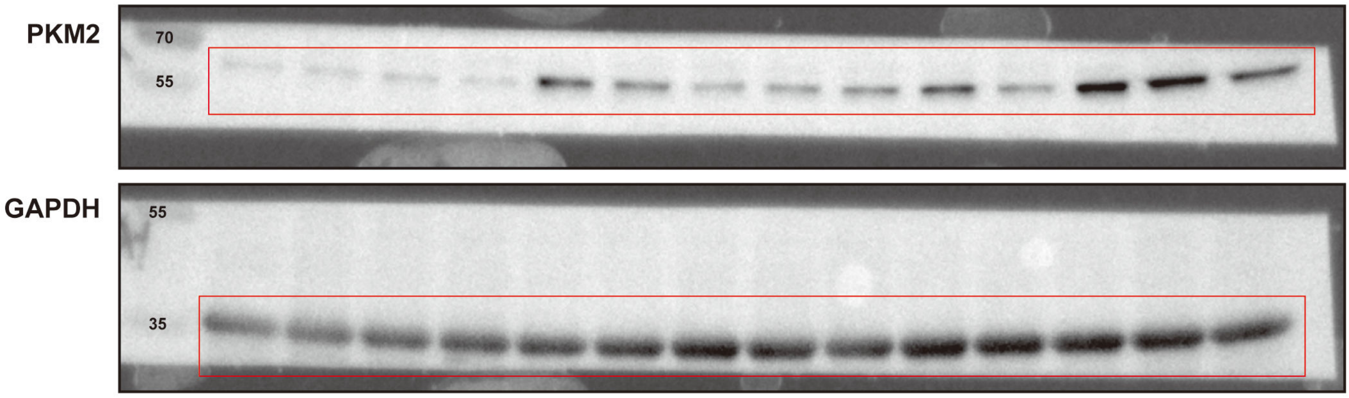

Figure 3H

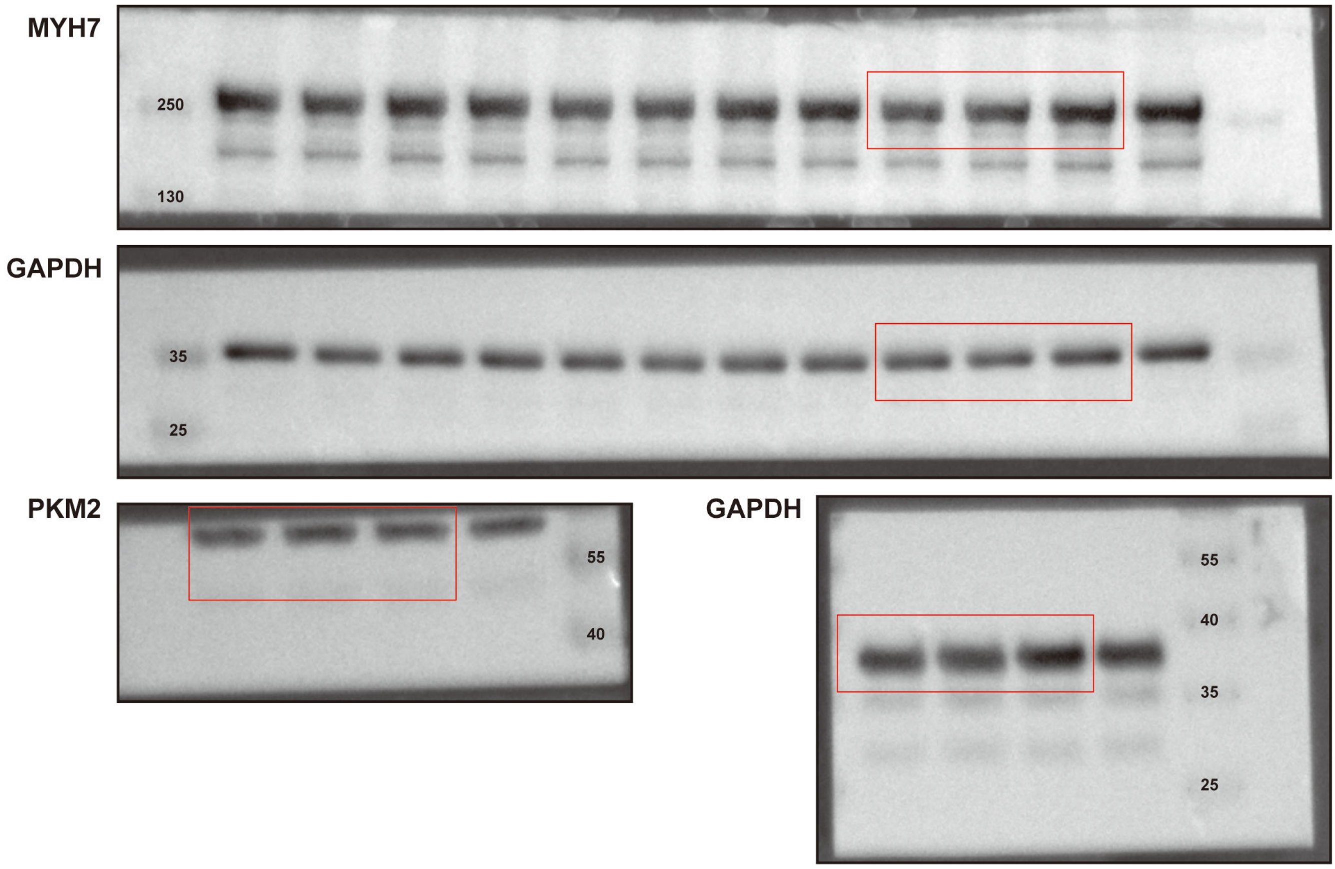

Figure 3K

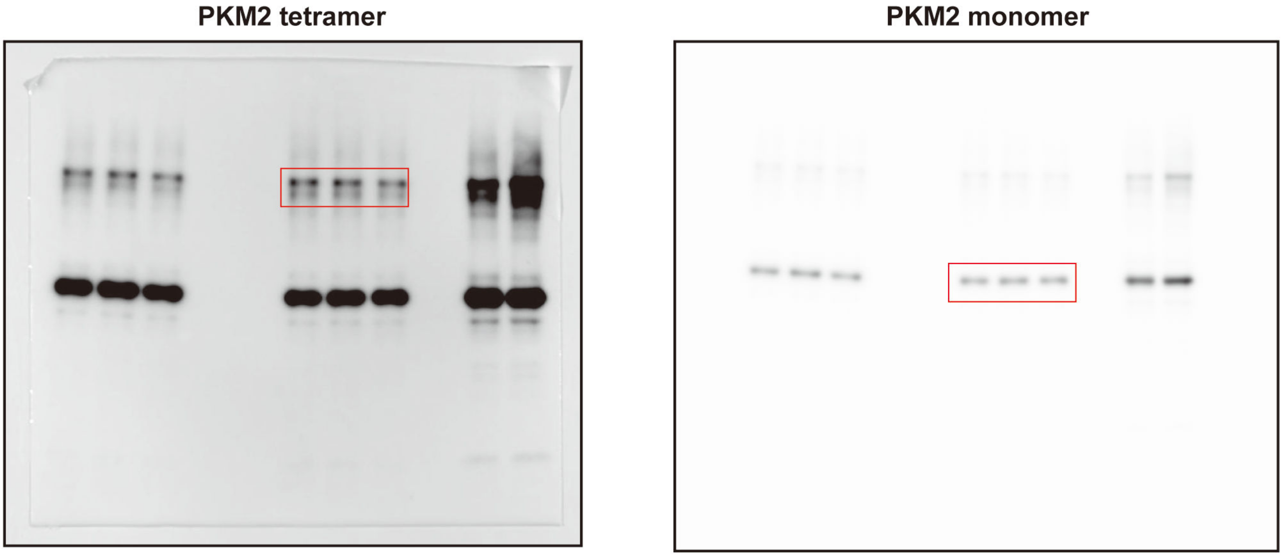

Figure 3M

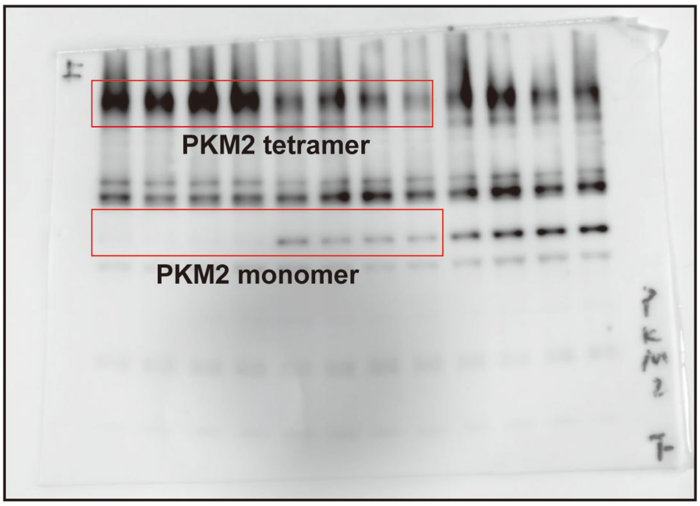

Figure 3P

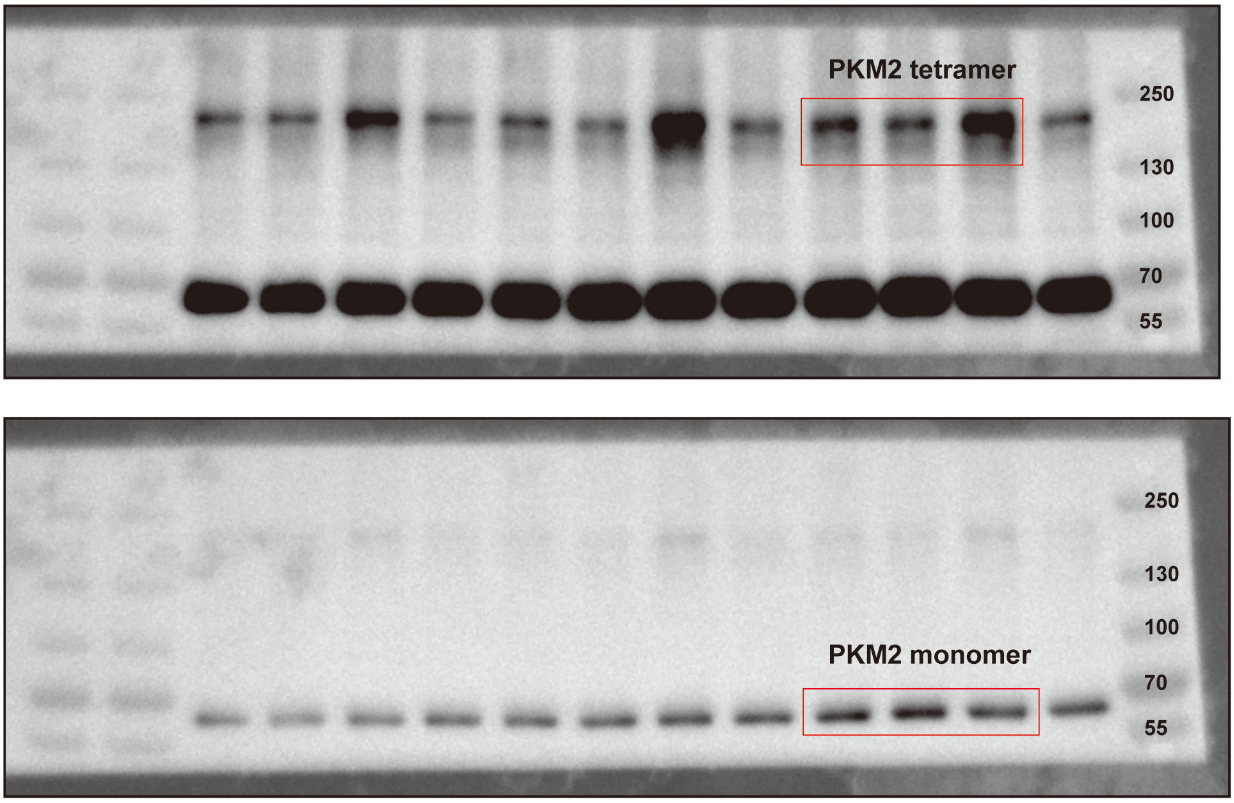

Figure 3K

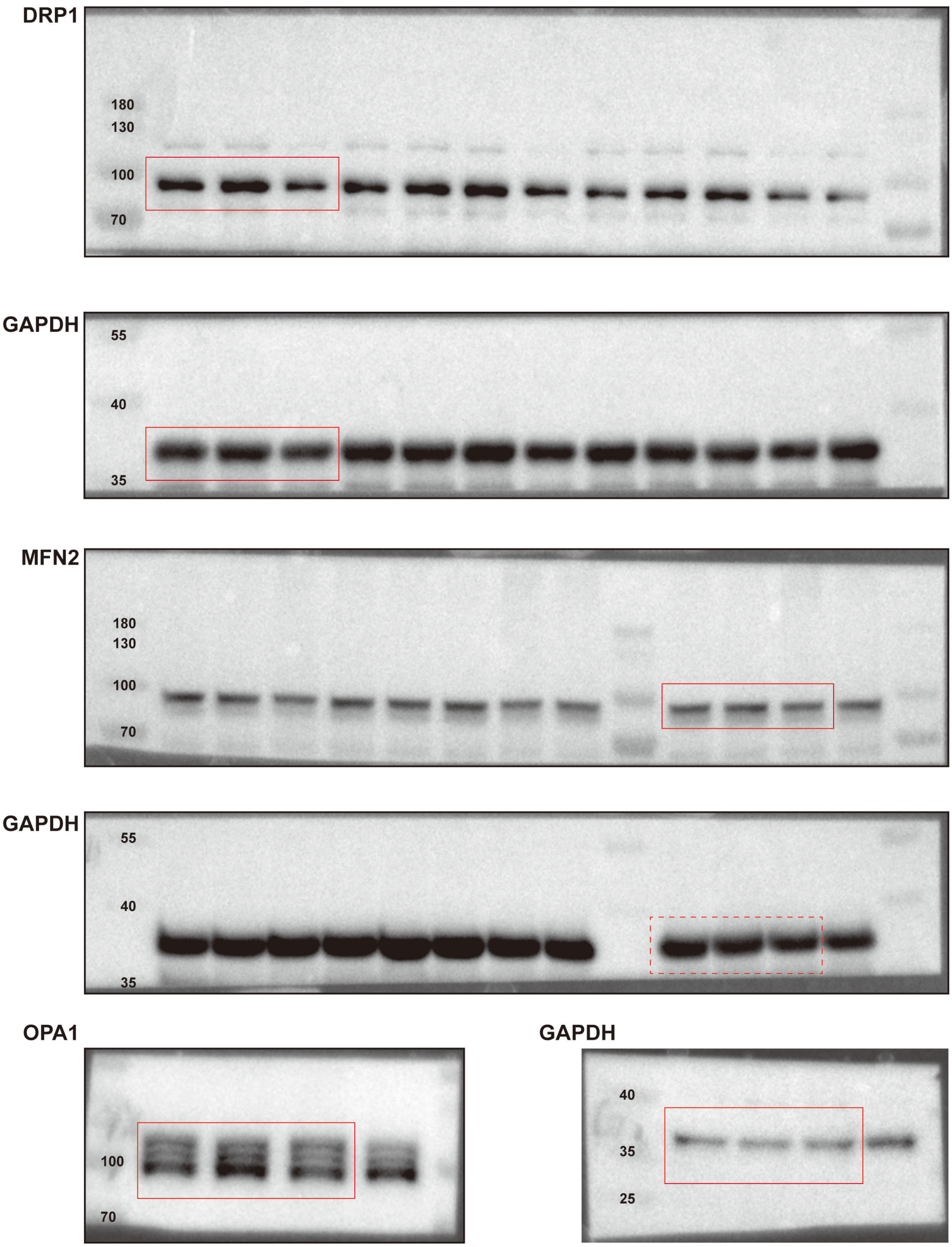
DRP1
180
130
100
70
GAPDH
55
40
35
MFN2
180
130
100
70
GAPDH
55
40
35
OPA1
100
70
GAPDH
40
35
25

Figure 5B

PKM2

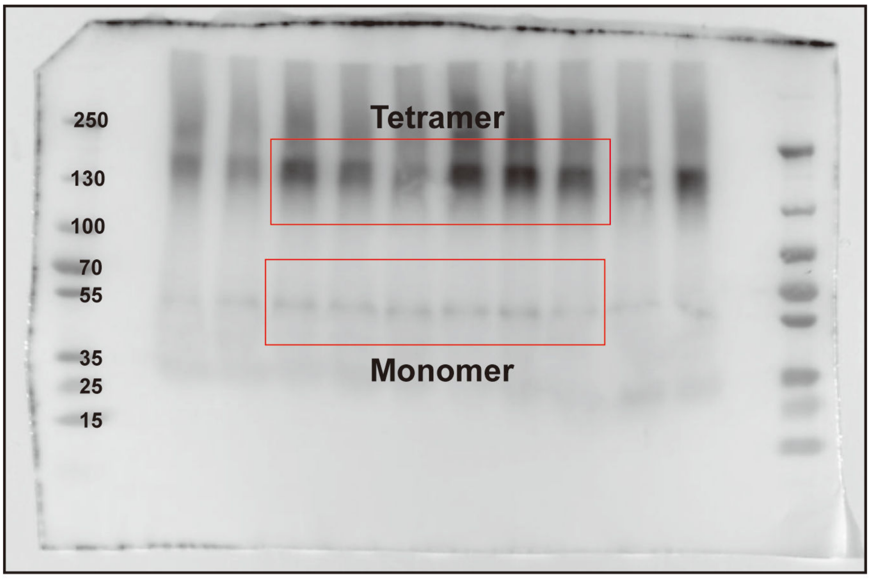

Figure 5J

Bcl-2

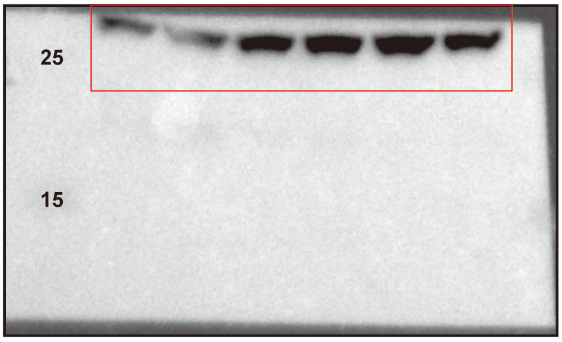

BAX

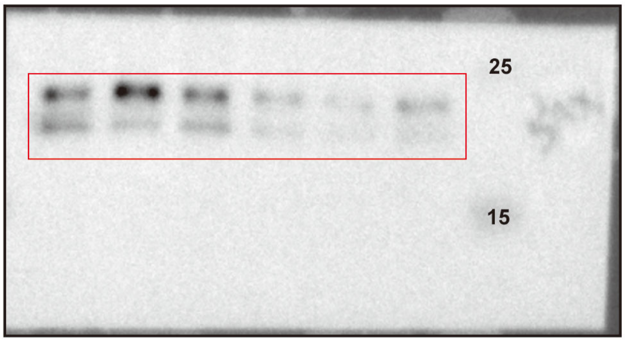

Caspase 7

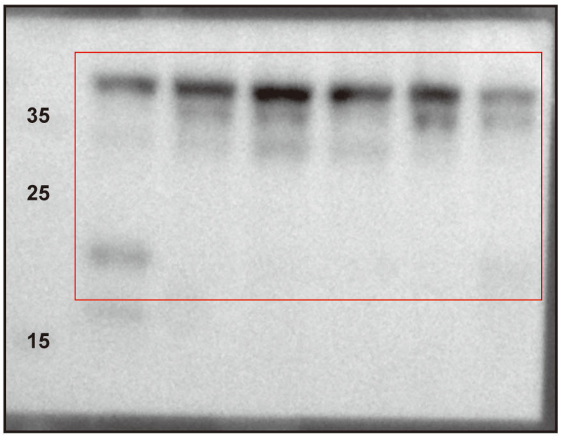

Caspase 3

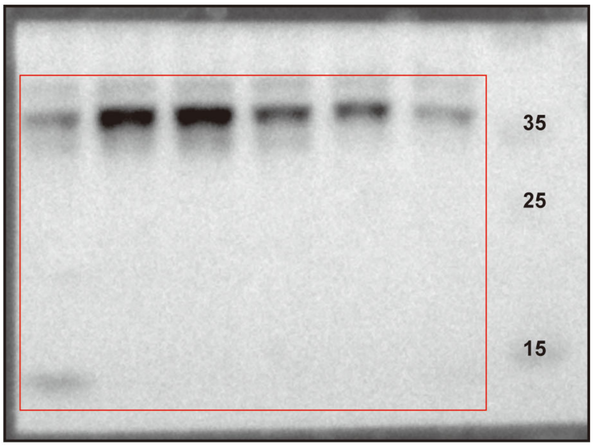

GAPDH

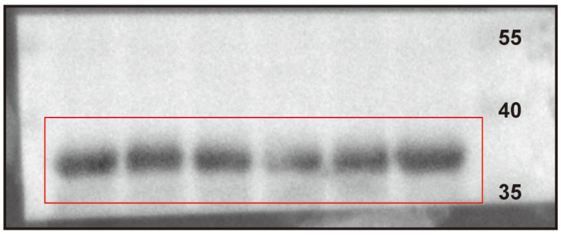

Supplementary figure 1F

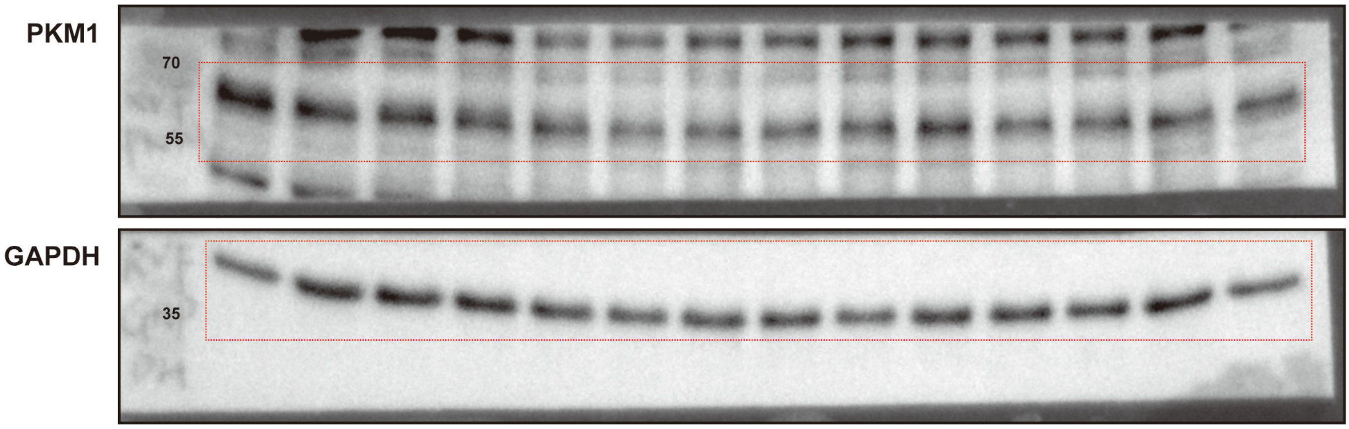

Supplementary figure 2F

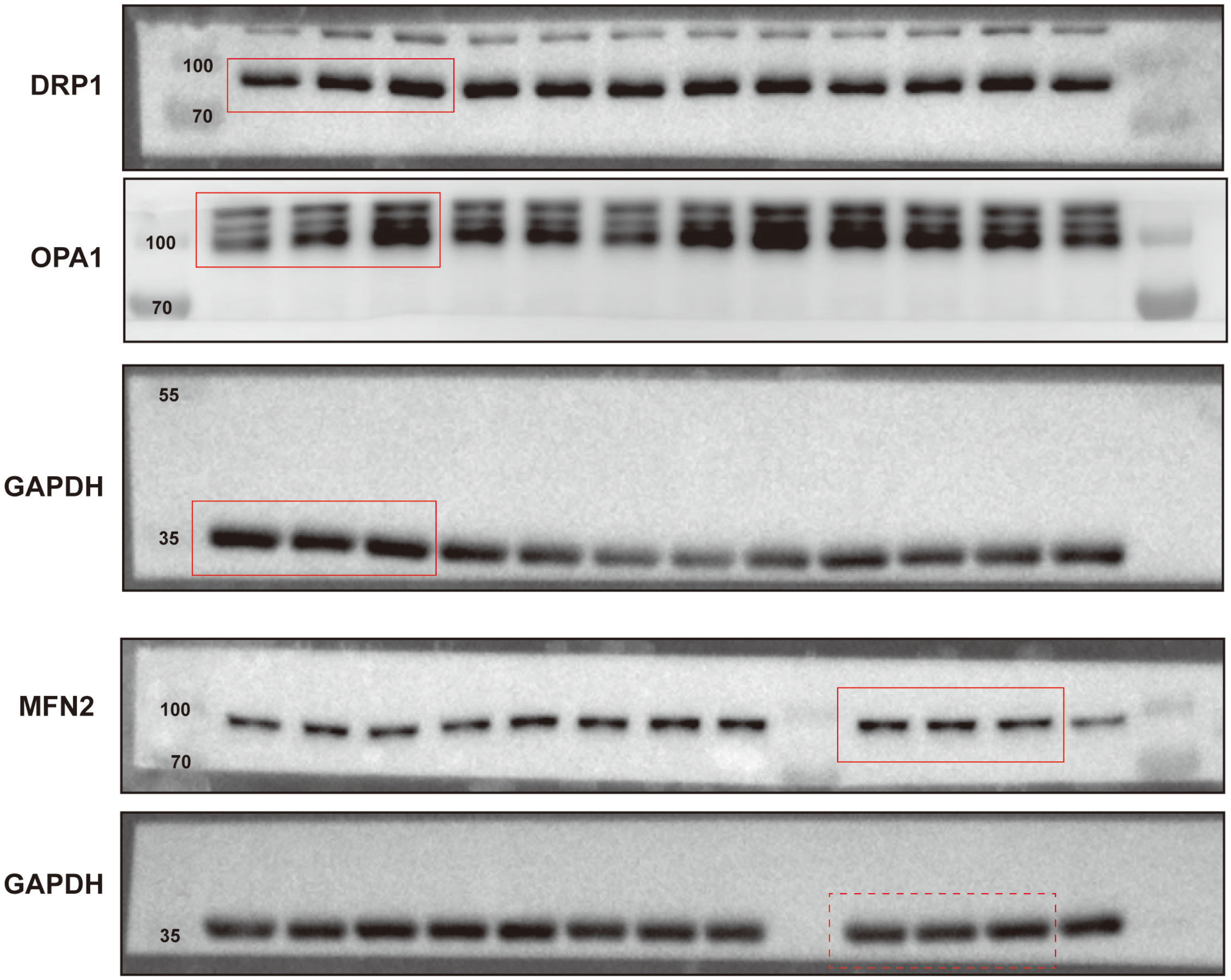

Supplement: Supplementary file 6 — Additional file 6: Uncropped gels from figures. [file 12967_2023_4780_MOESM6_ESM.pdf]
